# A type IV Autotaxin inhibitor ameliorates acute liver injury and nonalcoholic steatohepatitis

**DOI:** 10.15252/emmm.202216333

**Published:** 2022-07-14

**Authors:** Richell Booijink, Fernando Salgado‐Polo, Craig Jamieson, Anastassis Perrakis, Ruchi Bansal

**Affiliations:** ^1^ Translational Liver Research, Department of Medical Cell BioPhysics, Faculty of Science and Technology University of Twente Enschede The Netherlands; ^2^ Oncode Institute, Division of Biochemistry Netherlands Cancer Institute Amsterdam The Netherlands; ^3^ Department of Pure and Applied Chemistry University of Strathclyde Glasgow UK

**Keywords:** Autotaxin–lysophosphatidic acid (ATX‐LPA) axis, inflammation, fibrosis, liver disease, signaling pathways, Digestive System, Pharmacology & Drug Discovery

## Abstract

The lysophosphatidic acid (LPA) signaling axis is an important but rather underexplored pathway in liver disease. LPA is predominantly produced by Autotaxin (ATX) that has gained significant attention with an impressive number of ATX inhibitors (type I‐IV) reported. Here, we evaluated the therapeutic potential of a (yet unexplored) type IV inhibitor, Cpd17, in liver injury. We first confirmed the involvement of the ATX‐LPA signaling axis in human and murine diseased livers. Then, we evaluated the effects of Cpd17, in comparison with the classic type I inhibitor PF8380, *in vitro*, where Cpd17 showed higher efficacy. Thereafter, we characterized the mechanism‐of‐action of both inhibitors and found that Cpd17 was more potent in inhibiting RhoA‐mediated cytoskeletal remodeling, and phosphorylation of MAPK/ERK and AKT/PKB. Finally, the therapeutic potential of Cpd17 was investigated in CCl_4_‐induced acute liver injury and diet‐induced nonalcoholic steatohepatitis, demonstrating an excellent potential of Cpd17 in reducing liver injury in both disease models *in vivo*. We conclude that ATX inhibition, by type IV inhibitor in particular, has an excellent potential for clinical application in liver diseases.

## Graphical abstract

The type IV ATX inhibitor Cpd17 hinders the ATX‐LPA signaling axis by abrogating RhoA‐mediated cytoskeletal remodeling and phosphorylation of MAPK and AKT. Cpd17 also ameliorated induced liver injury in mice, showing a potential for clinical application of type IV ATX inhibitors in liver disease.

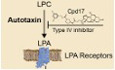

## Introduction

Liver diseases are an immense burden on today's modern society. Globally, millions of people suffer from liver diseases, causing approximately 2 million deaths per year (Asrani *et al*, 2019). Among several etiological liver diseases, drug‐induced acute liver injury (Bernal *et al*, 2010) and nonalcoholic fatty liver disease (NAFLD) and its severe form nonalcoholic steatohepatitis (NASH) have been recognized as a major public health concern in Western countries (Marcellin & Kutala, 2018; Younossi *et al*, 2018).

Liver injury caused by different factors, such as drug intoxication, excessive fat and/or alcohol intake, and hepatitis B/C viral infections, triggers hepatocyte injury and necrosis. This injury is accompanied by endoplasmic reticulum (ER) stress, oxidative stress through reactive oxygen species, and release of several pro‐inflammatory cytokines and chemokines (Byrne & Targher, 2015). Ongoing inflammation induces the release of pro‐fibrogenic mediators, mainly by resident and infiltrating macrophages (Kazankov *et al*, 2019), causing quiescent hepatic stellate cells (HSCs) to transdifferentiate into highly proliferative and activated HSCs (or myofibroblasts). These activated HSCs initiate a wound healing response that is characterized by the accumulation of an excessive extracellular matrix (ECM) at the injured site. Upon persistent liver injury, chronic inflammation, and ECM accumulation lead to liver fibrosis (scarring), cirrhosis (end‐stage liver dysfunction) (Byrne & Targher, 2015; Francque *et al*, 2016), and/or hepatocellular carcinoma (primary liver cancer) (Anstee *et al*, 2019).

Among several pathways that have been associated with liver diseases is the lysophosphatidic acid (LPA) signaling axis (Konerman *et al*, 2018). Besides liver diseases, the LPA signaling pathway is also involved in pathological conditions that include chronic inflammatory disorders, fibrotic diseases, and tumor progression (Choi *et al*, 2010; Balupuri *et al*, 2018). Recently, the ATX‐LPA signaling axis has also been shown to impede antitumor immunity by suppressing chemotaxis and tumor infiltration of CD8^+^ T cells (preprint: Matas‐Rico *et al*, 2021). The ATX/LPA pathway also plays a role in a plethora of biological processes including neurogenesis, vascular homeostasis, skeletal development and remodeling, and lymphocyte homing (Tigyi & Parrill, 2003; van Meeteren & Moolenaar, 2007; Kano *et al*, 2014; Sheng *et al*, 2015).

LPAs are physiologically occurring, structurally simple water‐soluble glycerol lysophospholipids that differ in length, and the number of saturated and unsaturated bonds of their alkyl chain. Their activity is exerted via the activation of downstream signaling pathways through G‐protein‐coupled receptors (GPCRs) specific for LPA. To date, six LPA receptors have been identified, that belong to the endothelial differentiation gene (EDG) family (LPA_1_–LPA_3_) and the P2Y family (LPA_4_–LPA_6_) (Mutoh *et al*, 2012), and couple with different Gα subunits upon activation. LPA receptor activation can occur through Gα(i/o) and Gα(12/13) driven cascades via phosphatidylinositol 3‐kinase (PI3K) and RhoA, respectively, resulting in cell survival and cytoskeletal remodeling, respectively (Teo *et al*, 2009; Yung *et al*, 2014).

The majority of LPA in the blood is synthesized from lysophosphatidylcholine (LPC) through hydrolysis of its choline moiety by Autotaxin (ATX), a member of the ectonucleotide pyrophosphatase phosphodiesterase family of enzymes (thus also known as ENPP2) (Borza *et al*, 2022). ATX is a multifunctional and multidomain protein that possesses enzymatic lysophospholipase D (lysoPLD) activity (Hausmann *et al*, 2011; Moolenaar & Perrakis, 2011). Structurally, ATX has a catalytic phosphodiesterase (PDE) domain, which accommodates a tripartite binding site: a hydrophilic shallow groove adjacent to the catalytic site that harbors the glycerol moiety of the lysolipid substrate; a hydrophobic pocket that binds the acyl chain; and a tunnel leading to the other side of the PDE domain (Hausmann *et al*, 2011). Given the emerging association with diseases, ATX has gained significant pharmacological and pharmacochemical attention (Geraldo *et al*, 2021), and an impressive number of ATX inhibitors has been reported (Salgado‐Polo & Perrakis, 2019). The only inhibitor to reach phase 3 clinical trial (for idiopathic pulmonary fibrosis) has been the GLPG1690 molecule (Zulfikar *et al*, 2020), a so‐called type IV inhibitor. Type IV inhibitors occupy the tunnel and the pocket of ATX, in contrast with type I inhibitors e.g., PF8380, which targets the catalytic site and the pocket (Salgado‐Polo & Perrakis, 2019).

Elevated ATX levels were observed in patients with different etiological liver diseases that correlated with disease severity including nonalcoholic fatty liver disease (NAFLD) (Nakagawa *et al*, 2011; Kondo *et al*, 2014; Yamazaki *et al*, 2017; Ando *et al*, 2018; Fujimori *et al*, 2018; Joshita *et al*, 2018). Moreover, hepatocyte‐specific genetic deletion of ATX resulted in abrogated liver damage, inflammation and diminished fibrosis, and deregulated fatty acid metabolism, thereby attenuating HCC development suggesting ATX as an attractive drug candidate in liver fibrosis and cancer (Kaffe *et al*, 2017). ATX pharmacological inhibition in liver disease has been evaluated in only a few preclinical studies, with varying outcomes. The type I inhibitor PF8380 was found to reduce fibrosis, but not inflammation and necrosis, in a CCl_4_‐induced liver fibrosis mouse model (Kaffe *et al*, 2017). An ATX type III inhibitor, PAT‐505, showed significant reduction of fibrosis score in two NASH mouse models, but no significant effect on inflammation, ballooning, or steatosis (Bain *et al*, 2017). The ATX inhibitor EX_31, a tetrahydrocarboline derivative with unclear binding mode, did not show any effect on liver‐related markers in a 10‐week CCl_4_‐induced liver fibrosis and 14‐week choline‐deficient amino acid‐defined diet (CDAA)‐induced NASH rat models (Baader *et al*, 2018). Interestingly, type IV ATX inhibitors have not yet been explored in liver diseases.

We have recently shown that mechanistic differences between type I and type IV inhibitors, rather than inhibitor potency in preventing the enzymatic hydrolysis of LPC to LPA alone, can lead to different physiological activity (preprint: Salgado‐Polo *et al*, 2022). Here, we set out to examine the effect of type IV inhibitor in liver disease models, as, despite the clear involvement of the ATX/LPA signaling pathway in liver diseases, inhibition of ATX has been underexplored in relevant liver disease models. We started by comparing the effects of our previously reported type IV Cpd17 inhibitor (Keune *et al*, 2017) (not yet explored in liver diseases), with the classic type I inhibitor PF8380 *in vitro*. Cpd17, designed by fusing parts of type I and type III inhibitors, occupies the binding pocket and the tunnel but does not interact with the catalytic site. While both Cpd17 and PF8380 possess similar potency in inhibiting LPC 18:0 to LPA conversion, our results demonstrate that Cpd17 exhibits higher potency in inhibiting downstream LPA signaling pathways *in situ* and *in vitro*. We were thus encouraged to examine Cpd17 in CCl_4_‐induced acute liver injury, and methionine‐ and choline‐deficient (MCD) diet‐induced NASH mouse models.

## Results

### Upregulation of Autotaxin in nonalcoholic steatohepatitis and liver cirrhosis

**Figure 1:**
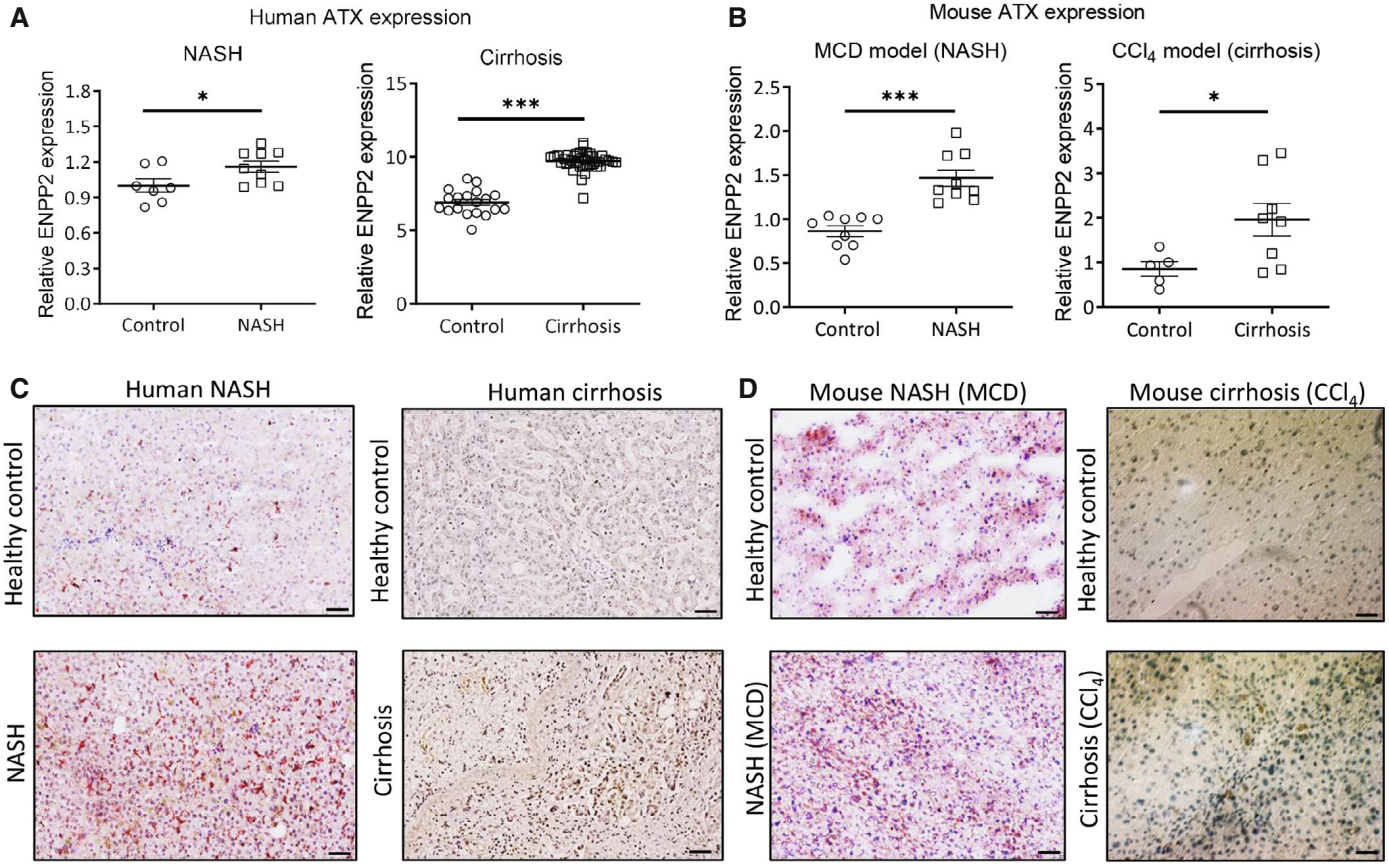
ATX (ENPP2) expression is upregulated in human and mouse livers AATX gene expression from publicly available datasets in NASH patients (*n* = 9) compared with healthy controls (*n* = 7) (*P < 0.05); cirrhosis patients (*n* = 40) compared with healthy controls (*n* = 19) (***P < 0.001). Bars represent the mean ± SEM, statistical analysis was performed by two‐tailed Student's *t*‐test. *P < 0.05, ***P < 0.001 denotes significance versus respective healthy controls.BRelative ATX gene expression in the MCD‐diet‐fed (*n* = 9) compared with control mice (*n* = 9) (***P < 0.001); CCl_4_ (*n* = 8) compared with control mice (*n* = 5) (*P < 0.05). Bars represent the mean ± SEM, statistical analysis was performed by two‐tailed Student's *t*‐test. *P < 0.05, ***P < 0.001 denotes significance versus respective healthy controls.CRepresentative images (scale = 50 μm) of ATX stained liver sections from human NASH (*n* = 5) compared to healthy controls (*n* = 4); human cirrhosis (*n* = 5) compared with healthy controls (*n* = 4).DRepresentative images (scale = 50 μm) of ATX stained liver sections (using AEC chromogen denoted by red color staining or DAB chromogen denoted by brown color staining; nuclei were stained blue with hematoxylin) from MCD‐diet‐fed NASH (*n* = 9) and CCl_4_‐induced liver fibrosis (*n* = 8) mouse models compared with respective controls (*n* = 9 or *n* = 5). Source data are available online for this figure.

Upregulation of ATX in chronic liver diseases, including NASH and liver cirrhosis, has been previously observed in animal models, and in human patients (Kaffe *et al*, 2017; Fujimori *et al*, 2018). To confirm these findings, ATX (ENPP2) gene expression has been analyzed in human and mice livers. Analysis of mRNA expression levels revealed significantly elevated ENPP2/ATX expression in human NASH patients, compared with healthy control livers (Fig 1A). Similar upregulation of ATX gene expression was also observed in patients with liver cirrhosis (Fig 1A). Consistent with human data, ATX gene expression in mouse livers was upregulated in the MCD diet‐induced NASH mouse model and in our CCl_4_‐induced liver cirrhosis mouse model (Fig 1B).

We further examined the protein expression of ATX in human and mouse livers by immunostaining. Hereby, we also found an increase in ATX protein expression in human NASH and cirrhotic livers compared with normal livers (Fig 1C). As expected, this was confirmed in both MCD‐induced NASH and CCl_4_‐induced cirrhosis mouse models compared with respective healthy controls (Fig 1D).

### Inhibition of ATX in hepatocytes and LPS/IFNγ‐activated pro‐inflammatory macrophages

**Figure 2:**
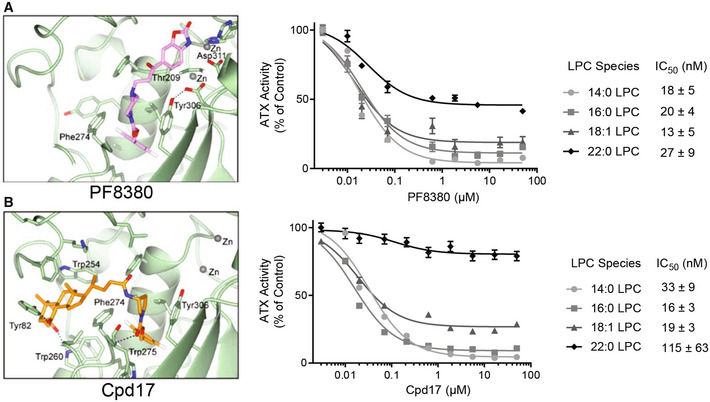
ATX inhibitors PF8380 and Cpd17 decrease LPA production AStructural binding of type I ATX inhibitor PF8380 to ATX; Potency (IC_50_) of PF8380 for inhibiting the catalysis of LPC to LPA. Bars represent the mean + SEM, *n* = 3.BStructural binding of type IV ATX inhibitor Cpd17 to ATX; Potency (IC_50_) of Cpd17 for inhibiting the catalysis of LPC to LPA. Bars represent the mean + SEM, *n* = 3.

**Figure EV1:**
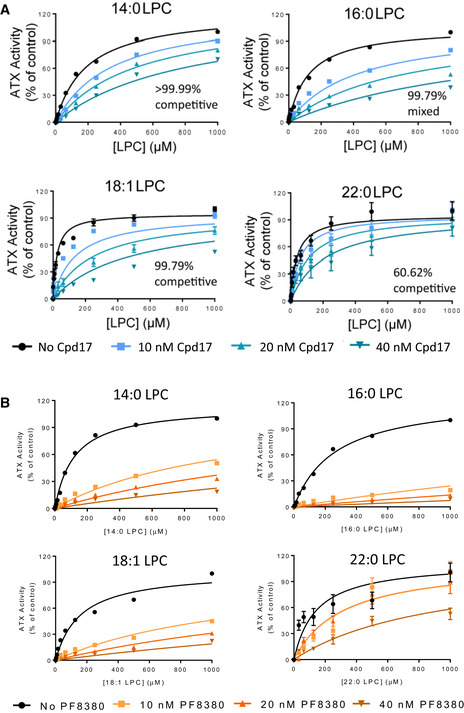
Cpd17 and PF8380 inhibit ATX activity over different LPC species A, BCpd17 (A) and PF8380 (B) were incubated with 20nM ATX for at least 30 min, upon which mixes were added to plates containing LPC and the reaction buffer for detection of choline release. Reaction kinetics were followed over time. Activity slopes were quantitated by linear regression and plotted versus LPC concentration. Bars represent the Mean ± SEM, *n* = 3. Quantitation of the type of inhibition was performed by comparison of non‐linear regressions to competitive and non‐competitive modes of inhibition.

To inhibit the function of ATX, and thus the production of LPA and the related downstream signaling, we focused on the well‐characterized type I inhibitor PF8380 (Fig 2A) and our type IV inhibitor Cpd17 (Fig 2B). These two inhibitors possess similar potency in inhibiting the catalysis of LPC to LPA but distinct binding modes on the ATX tripartite binding site. Their effect on LPC species that we tested was similar (for 14:0, 16:0, and 18:1 LPC species) but showed interesting differences with Cpd17 being less potent in ameliorating hydrolysis of longer alkyl chain LPC species particularly 22:0 LPC (Figs 2 and EV1). We have thus decided to check both inhibitors in specific assays *in vitro* to evaluate their therapeutic potential.

**Figure 3:**
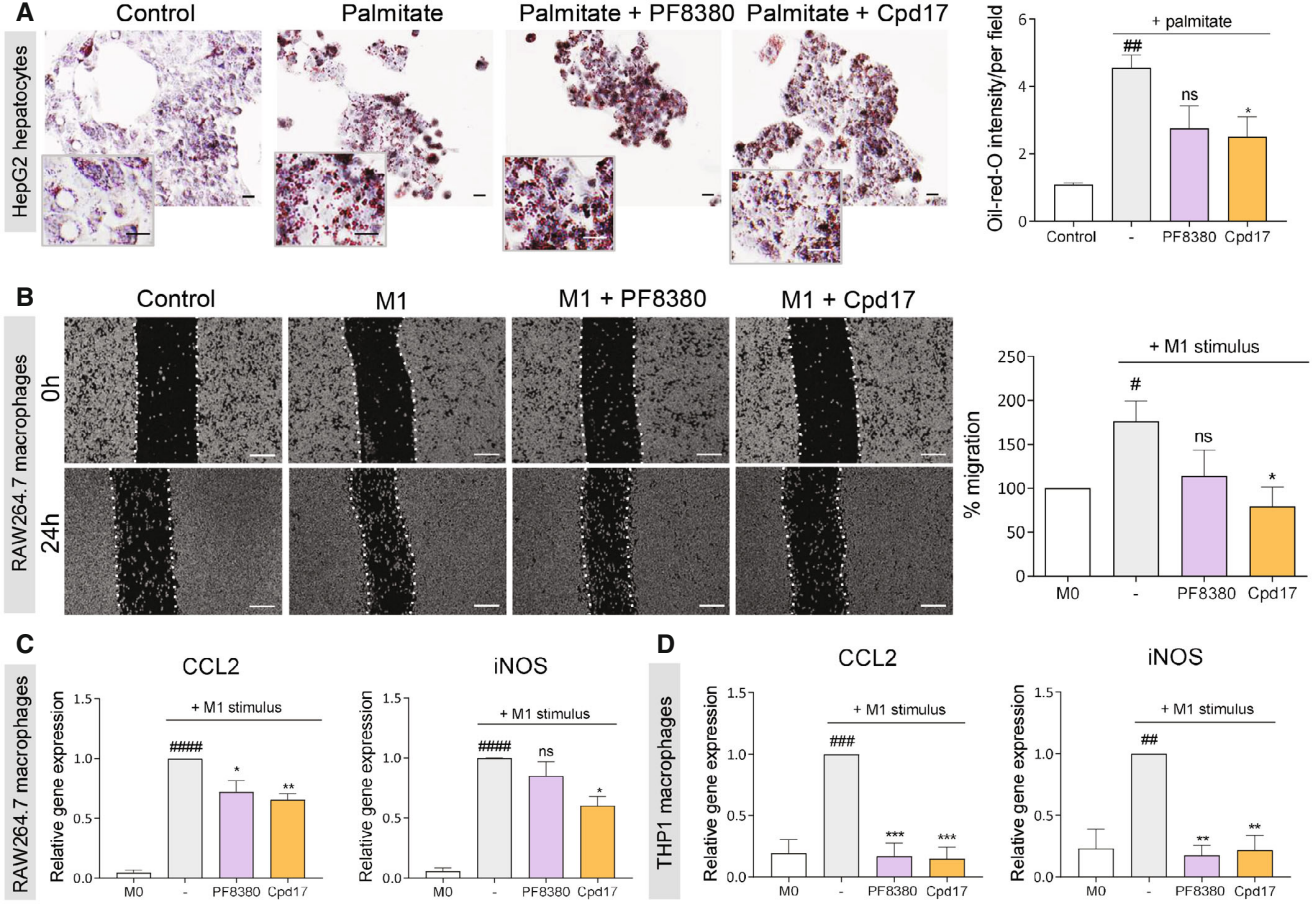
ATX inhibitors decrease steatosis in hepatocytes, and migration and expression of inflammation‐related genes in macrophages ARepresentative images (scale = 10 μm) and quantification of HepG2 cells stained with oil‐red‐O after 48 h of incubation with or without 200 μM palmitate with or without 1 μM of PF8380 or Cpd17. Bars represent the mean + SEM, *n* = 3. Statistical analysis was performed by one‐way analysis of variance (ANOVA) with Dunnett's multiple comparison test. ^##^ *P* < 0.01 denotes significance versus control (non‐palmitate) and *P < 0.05 denotes significance versus vehicle (palmitate); ns = nonsignificant.BRepresentative images (at 0 h and 24 h) (scale = 50 μm) and quantitative analysis (after 24 h) of migration by control (M0) and LPS‐ and IFNγ‐induced M1 RAW264.7 macrophages treated with medium alone, PF8380 (1 μM) or Cpd17 (1 μM). Bars represent the mean + SEM, *n* = 4. Statistical analysis was performed by one‐way analysis of variance (ANOVA) with Bonferroni *post hoc* test. ^#^ *P* < 0.05 denotes significance versus M0 (nonstimulated macrophages) and *P < 0.05 denotes significance versus vehicle (LPS‐ and IFNγ‐induced M1 macrophages); ns = nonsignificant.CRelative gene expression (normalized with GAPDH) for CCL2 and iNOS in control (M0) and LPS‐ and IFNγ‐induced M1 RAW 264.7 macrophages treated with medium alone, PF8380 (1 μM) or Cpd17 (1 μM). Bars represent the mean + SEM, *n* = 4. Statistical analysis was performed by one‐way analysis of variance (ANOVA) with Bonferroni *post hoc* test. ^####^ *P* < 0.0001 denotes significance versus M0 (nonstimulated macrophages) and *P < 0.05, **P < 0.01 denotes significance versus vehicle (LPS‐ and IFNγ‐induced M1 macrophages); ns = nonsignificant.DRelative gene expression (normalized with 18 s RNA) for CCL2 and iNOS in control (M0) and LPS‐induced PMA‐differentiated human THP1 macrophages treated with medium alone, PF8380 (1 μM) or Cpd17 (1 μM). Bars represent the mean + SEM, *n* = 4. Statistical analysis was performed by one‐way analysis of variance (ANOVA) with Bonferroni *post hoc* test. ^##^ *P* < 0.01, ^###^ *P* < 0.001 denotes significance versus M0 (nonstimulated macrophages) and **P < 0.01, ***P < 0.001 denotes significance versus vehicle (LPS‐ and IFNγ‐induced M1 macrophages); ns = nonsignificant. Source data are available online for this figure.

Previously, it has been reported that different hepatotoxic stimuli stimulate hepatocyte ATX expression, leading to activation of fibrogenic pathways. Furthermore, hepatocyte‐specific ablation and/or transgenic overexpression of ATX suggested a role of ATX/LPA in liver cirrhosis and HCC (Kaffe *et al*, 2017). Moreover, as ATX has shown to be involved in fatty acid metabolism (Kaffe *et al*, 2017), in this study, we first investigated the implication of pharmacological ATX inhibition using two different ATX inhibitors (Cpd17 and PF8380) in free fatty acid (Palmitate)‐treated hepatocytes. To mimic NASH phenotype, human hepatoblastoma cell line, HepG2 cells, were exposed to pathophysiological relevant concentrations of palmitic acid to mimic excessive influx of fatty acids into hepatocytes (Joshi‐Barve *et al*, 2007) and found that ATX inhibition by Cpd17 significantly reduced lipid accumulation in hepatocytes compared with PF8380 as assessed using Oil‐red‐O staining (Fig 3A).

Following hepatocellular damage, the inflammatory response plays a key role in the initiation and progression of liver fibrosis. Upon liver injury, infiltrating monocytes undergo differentiation into traditionally defined classically activated pro‐inflammatory (M1) macrophages, which mediate the initial inflammatory response, while alternatively activated pro‐resolving (M2) macrophages predominantly mediate the resolution phase (Seki & Schwabe, 2015). We thus investigated the effect of the PF8380 and Cpd17 in classically activated mouse RAW264.7 macrophages and PMA‐differentiated human THP1 macrophages. To investigate the effect of ATX inhibition on the migration of pro‐inflammatory M1 (activated by IFN‐γ and LPS) RAW264.7 macrophages, we performed a scratch assay. 24 h after making a scratch, LPS/IFNγ‐activated pro‐inflammatory RAW264.7 macrophages showed a visibly significant increase in migration, compared with unpolarized (M0) macrophages (Fig 3B). Inhibition of ATX by PF8380 showed some (nonsignificant) decrease in macrophage migration; however, Cpd17 significantly reduced the percentage of cell migration compared with that of LPS/IFNγ‐activated macrophages (Fig 3B). We further examined the efficacy of the inhibitors on the gene expression of inflammatory markers in both mouse and human macrophages. In mouse RAW264.7 macrophages, we observed that both PF8380 and Cpd17 significantly alleviated the LPS/IFNγ‐induced expression of C‐C chemokine 2 (CCL2), also known as monocyte chemotactic protein 1 (MCP1), one of the key chemokines that regulate migration and infiltration of monocytes/macrophages. Moreover, Cpd17, but not PF8380, also alleviated the expression of other inflammatory marker inducible nitric oxide synthase (iNOS) (Fig 3C). Similarly, in human THP1 macrophages, M1 activation through LPS and IFNγ led to an increase in gene expression of inflammatory markers CCL2 and iNOS. After 24‐h treatment with both inhibitors, gene expression levels were significantly decreased (Fig 3D).

These results show that inhibition of ATX decreases lipid accumulation in hepatocytes and attenuates LPS/IFNγ‐induced pro‐inflammatory macrophage activation and migration, both in murine and in human cells. These results confirm the importance of the LPA/ATX pathway in liver inflammation. Furthermore, the results suggest that Cpd17 is more efficient than PF8380 in ameliorating induced steatosis and inflammatory phenotype.

### Inhibition of ATX in TGFβ‐activated hepatic stellate cells

**Figure 4:**
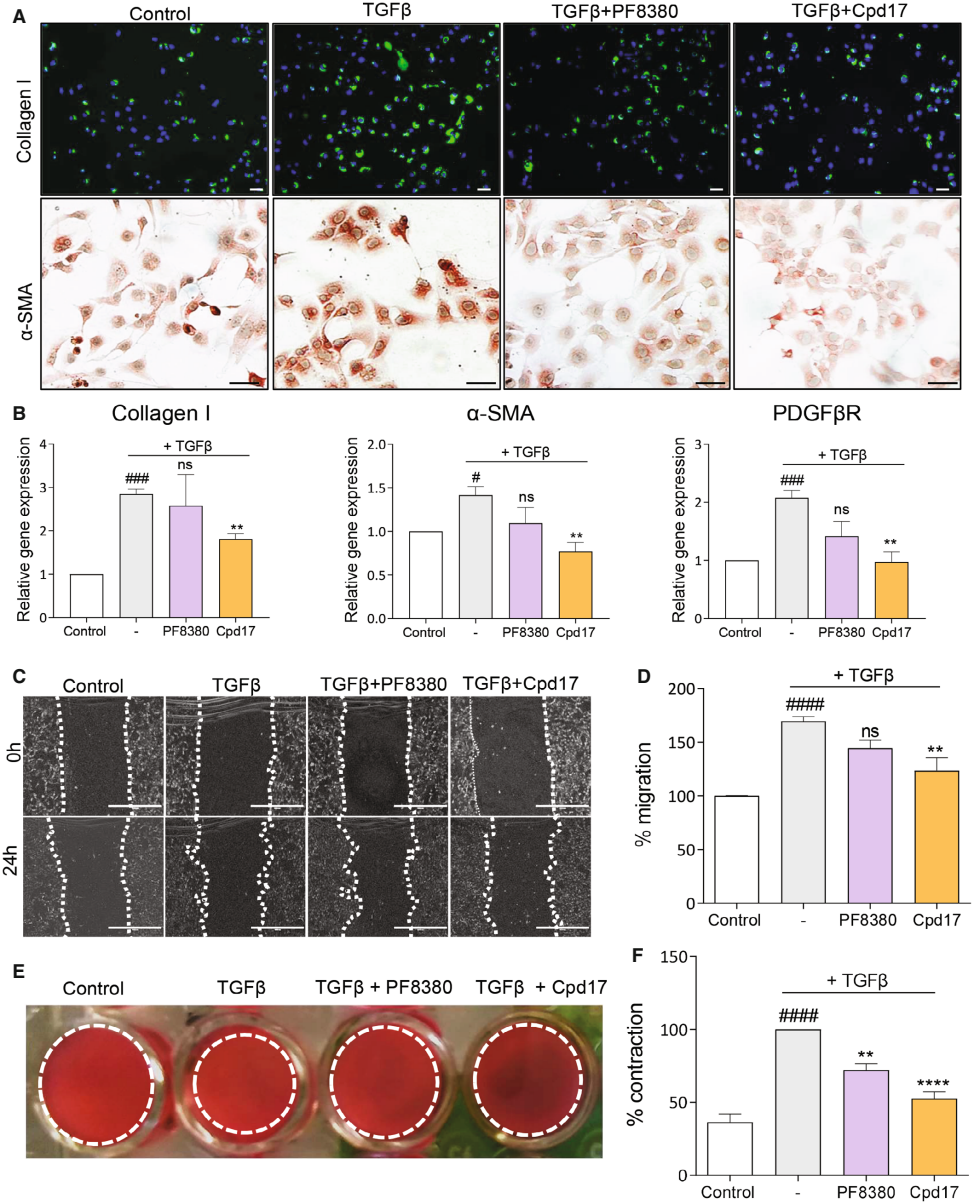
ATX inhibition decreases TGFβ‐induced HSCs activation, migration, and contraction ACollagen I and α‐SMA stained images (scale = 50 μm) of control and TGFβ‐activated LX2 cells with or without PF8380 (1 μM) or Cpd17 (1 μM) (*n* = 3).BRelative Collagen I, α‐SMA and PDGFβR gene expression (normalized with GAPDH) in control and TGFβ‐activated LX2 cells with or without PF8380 (1 μM) or Cpd17 (1 μM). Bars represent the mean + SEM, *n* = 3. Statistical analysis was performed by two‐tailed Student's *t*‐test. ^#^ *P* < 0.05, ^###^ *P* < 0.001 denotes significance versus control (non‐TGFβ cells) and **P < 0.01 denotes significance versus vehicle (TGFβ‐activated cells); ns = nonsignificant.C, DRepresentative images (at 0 h and 24 h) (scale = 100 μm) (C) and quantitative analysis (after 24 h) (D) of migration by control and TGFβ‐activated LX2 cells with or without PF8380 (1 μM) or Cpd17 (1 μM). Bars represent the mean + SEM, *n* = 3. Statistical analysis was performed by one‐way analysis of variance (ANOVA) with Bonferroni *post hoc* test. ^####^ *P* < 0.0001 denotes significance versus control (non‐TGFβ cells) and **P < 0.01 denotes significance versus vehicle (TGFβ‐activated cells); ns = nonsignificant.E, FRepresentative images (E) and quantitative analysis (F) showing 3D collagen gel contractility of control and TGFβ‐activated LX2 cells with or without PF8380 (1 μM) or Cpd17 (1 μM). Bars represent the mean + SEM, *n* = 3. Statistical analysis was performed by one‐way analysis of variance (ANOVA) with Bonferroni *post hoc* test. ^####^ *P* < 0.0001 denotes significance versus control (non‐TGFβ cells) and **P < 0.01, ****P < 0.0001 denotes significance versus vehicle (TGFβ‐activated cells). Source data are available online for this figure.

The elevated ATX gene expression in patients could be recapitulated upon the activation of fibrogenesis *in vitro* in HSCs. This observation strengthened the previously reported association between ATX expression and the development of liver fibrosis and offered an attractive system for *in vitro* studies. We therefore investigated the effect of PF8380 and Cpd17 in TGFβ‐activated LX2 cells (immortalized human HSCs). Upon TGFβ activation, immunostainings revealed an increase in fibrosis‐related proteins, collagen I (the major ECM protein) and α‐SMA (the HSCs activation marker) (Fig 4A); the addition of PF8380 or Cpd17 visibly decreased the protein levels (Fig 4A). Correspondingly, gene expression levels of collagen I, α‐SMA and platelet‐derived growth factor beta receptor (PDGFβR, involved in HSC proliferation and survival), increased significantly upon TGFβ activation, showed some (nonsignificant) decrease after PF8380 treatment, but stronger (significant) reduction after Cpd17 treatment (Fig 4B). During fibrogenesis, increase in liver stiffness and excessive ECM accumulation is mediated by HSCs differentiation into myofibroblasts and HSCs migration to the injured site (Zhang *et al*, 2016). The migratory potential of activated HSCs was evaluated by a scratch assay. As observed previously, Cpd17 significantly reduced the wound healing response (Fig 4C and D), indicating a reduction in the migratory properties of the LX2 cells. Finally, we studied the effect of the two ATX inhibitors on the contractile properties of the LX2 cells using a 3D‐collagen I contraction assay. Results showed that both PF8380 and Cpd17 significantly reduced TGFβ‐induced collagen I contraction of LX2 cells after 24‐h treatment (Fig 4E and F); consistent with other results the effect was more pronounced with Cpd17 treatment.

Overall, these results further solidify the significant role of the LPA/ATX pathway in TGFβ‐induced activation, migration, and contractility of human HSCs, and confirm that the ATX type IV inhibitor Cpd17 is more efficient in reducing the fibrotic effects caused by HSC activation.

### PF8380 and Cpd17 differentially inhibit downstream signaling in LX2 cells and RAW macrophages

**Figure 5:**
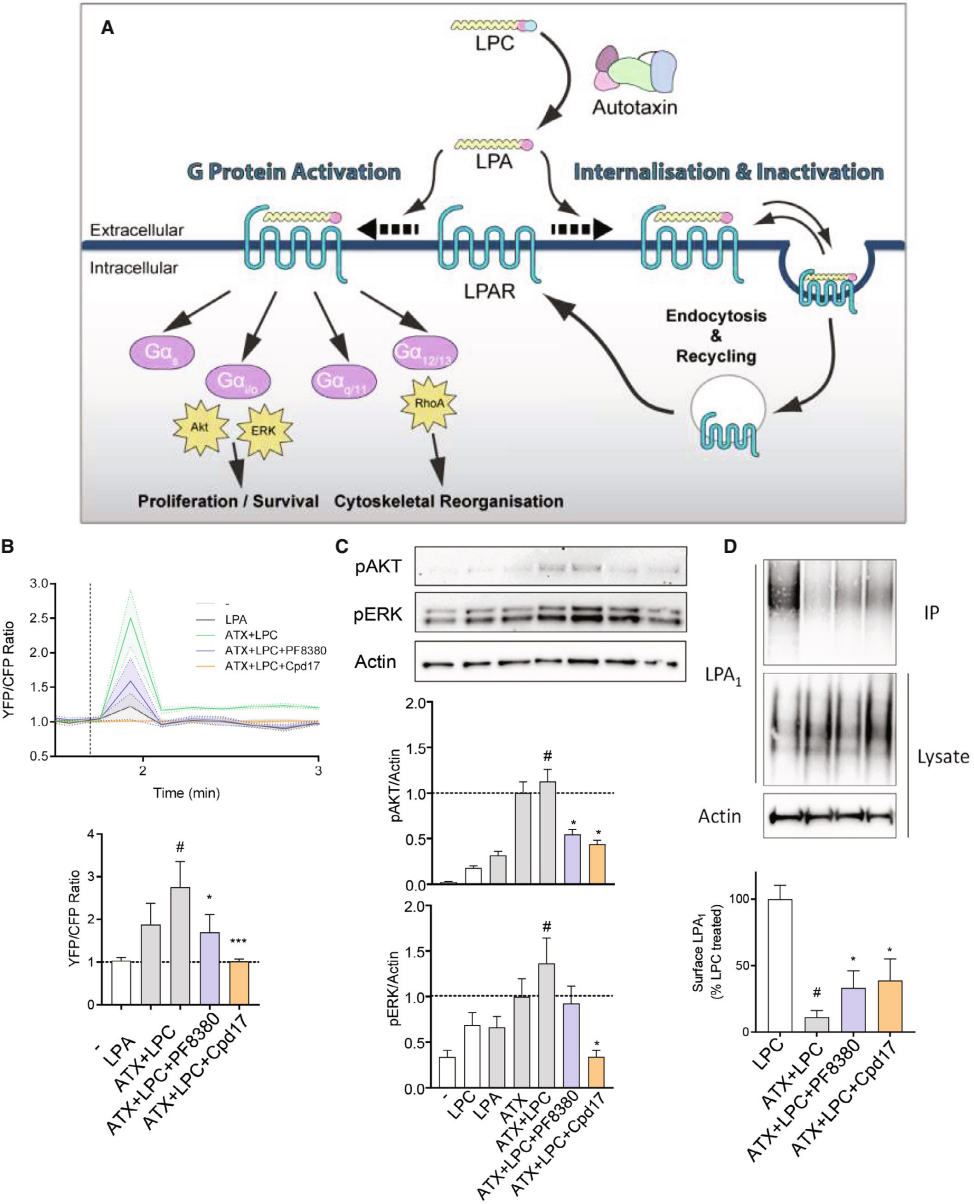
Inhibition of ATX results in blockade of LPAR‐driven activation of stellate cells and macrophages ASchematic overview of LPA‐induced signaling pathways.BTop panel shows time‐course stimulation to LPA and uninhibited or inhibited LPC‐treated ATX followed by RhoA activation, measured as a YFP/CFP fluorescent ratio. Bottom panel depicts quantitation of the burst response to the stimulants. Bars represent the Mean + SEM, *n* = 3. Statistical analysis was performed by one‐way analysis of variance (ANOVA) with Bonferroni *post hoc* test. ^#^ *P* < 0.05 denotes significance versus control (nonstimulated cells); *P < 0.05, ***P < 0.001 denotes significance versus ATX + LPC‐treated cells.CRepresentative images and quantification showing Western blot analysis for p‐Akt and p‐ERK normalized with β‐actin in control, LPC, LPA, ATX, ATX + LPC, ATX + LPC + PF8380, and ATX + LPC + Cpd17 stimulated LX2 cells. Bars represent the Mean + SEM, *n* = 3. Statistical analysis was performed by one‐way analysis of variance (ANOVA) with Bonferroni *post hoc* test. ^#^ *P* < 0.05 denotes significance versus control (nonstimulated cells); *P < 0.05 denotes significance versus ATX + LPC‐treated cells.DRepresentative images and quantification showing Western blot analysis for LPAR1 on the cell surface and in lysate, normalized with β‐actin in lysate, in LPC, ATX + LPC, ATX + LPC + PF8380, and ATX + LPC + Cpd17 stimulated LX2 cells. Bars represent the Mean + SEM, *n* = 3. Statistical analysis was performed by one‐way analysis of variance (ANOVA) with Bonferroni *post hoc* test. ^#^ *P* < 0.05 denotes significance versus control (nonstimulated cells); *P < 0.05 denotes significance versus ATX + LPC‐treated cells. Source data are available online for this figure.

**Figure EV2:**
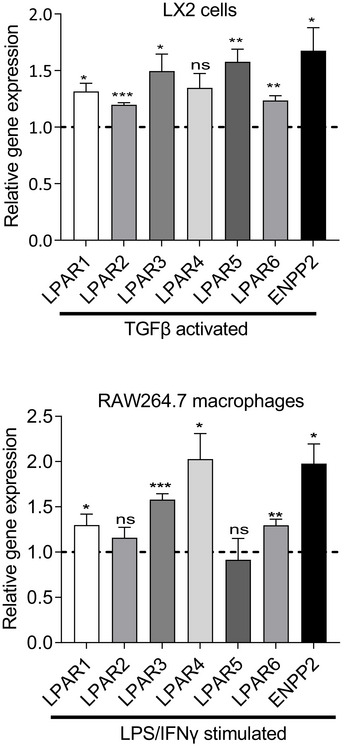
The ATX/LPA pathway related genes (ENPP2 and LPAR_1‐6_) are upregulated in activated LX2 cells and pro‐inflammatory RAW264.7 macrophages Relative LPAR_1‐6_ and ENPP2 gene expression (normalized with GAPDH) in TGFβ‐activated LX2 cells versus control (top) and in LPS/IFNγ stimulated M1 RAW264.7 macrophages versus control (bottom). Bars represent the Mean ± SEM, *n* = 3. Statistical analysis was performed by two‐tailed students *t*‐test with Welch's correction. Results are normalized with respective control (non‐activated/unstimulated) cells depicted by dashed lines in both graphs. *P < 0.05, **P < 0.01, ***P < 0.001 denotes significance versus control (non‐stimulated cells). ns = non‐significant.

To investigate the mechanism by which PF8380 and Cpd17 affect inflammatory and fibrotic responses, we sought to dissect their downstream signaling effects in macrophages and HSCs, respectively. A short overview of LPA‐induced signaling pathways is depicted in Fig 5A. As all downstream signaling depends on the levels of ATX and the LPA_1–6_ receptors, we first evaluated their gene expression levels in TGFβ activated LX2 cells and in LPS/IFNγ‐activated pro‐inflammatory RAW264.7 macrophages (Fig EV2). We observed that all the LPA receptors (except LPA_4_), as well as ENPP2, were upregulated in TGFβ‐activated LX2 cells; and all LPA receptors (except LPA_2_ and LPA_5_), and ENPP2 were significantly increased in LPS/IFNγ‐induced pro‐inflammatory RAW264.7 macrophages, consistent with existing literature (Kaffe *et al*, 2017; Gurvich *et al*, 2020; He *et al*, 2020; Wang *et al*, 2020) (Fig EV2).

**Figure EV3:**
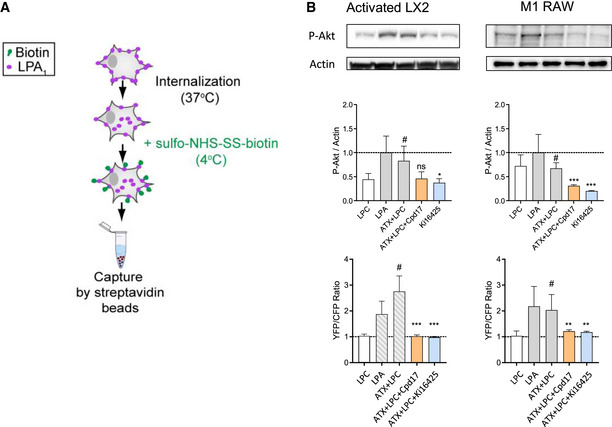
ATX inhibition by Cpd17 resembles antagonism of LPA_1_ by Ki16425 ASchematic procedure of LPA_1_ internalization assay for quantitation of surface localization. Data shown in Fig 5D.BRepresentative Western‐blot images and quantitation of pAKT normalized to β‐actin. Serum‐starved TGFβ‐activated LX2 cells and M1 (LPS + IFNγ)‐polarized RAW264.7 macrophages were treated for 10 min with the stimulants as indicated in the graphs. Bottom plots show the quantitation of RhoA activation from time‐course stimulation of activated and serum‐starved LX2 and M1‐polarized RAW264.7 macrophages. RhoA activation is measured as a YFP/CFP fluorescent ratio. Bars represent the Mean + SEM, *n* = 3. Statistical analysis was performed by one‐way analysis of variance (ANOVA) with Bonferroni *post‐hoc* test; ^#^ *P* < 0.05 denotes significance versus control (non‐stimulated cells); *P < 0.05, **P < 0.01, ***P < 0.001 denotes significance versus ATX + LPC treated cells. ns = non‐significant. Source data are available online for this figure.

With respect to the signaling pathways, we first monitored the Gα(12/13)‐mediated RhoA signaling pathway that leads to cytoskeletal reorganization, as it relates to HSCs morphology, migration, and contractility (Yanase *et al*, 2000), which are hallmarks for mediating the fibrotic effect in HSCs. Consistently with the phenotypic observations, we observed that Cpd17, but not PF8380, strongly inhibited the ATX + LPC(LPA)‐induced RhoA activation in LX2 cells (Fig 5B). We then checked the Gα(i/o)‐mediated phosphorylation of AKT and extracellular signal‐regulated kinase (ERK). The maximal response in the amount of pAKT and pERK in LX2 cells is upon stimulation with ATX and the LPC substrate; this effect was ameliorated by both inhibitors in the case of pAKT accumulation, but only Cpd17 significantly suppressed pERK (Fig 5C). We then checked the alternative signaling pathway involving receptor internalization, by biotin labeling of surface proteins, immunoprecipitation, and visualization of the amount of LPA_1_ in the cell surface (Fig EV3A). While treatment with ATX and LPC significantly decreased the amount of LPA_1_ receptor in the cell surface, both inhibitors partially restored LPA_1_ surface levels (Fig 5D). Finally, to confirm that the effects that we see are LPA‐receptor dependent, we used the Ki16425 antagonist, mostly acting on LPA_1_, and demonstrated that its application had effects similar to those of the ATX inhibitors, and particularly Cpd17, both in LX2 cells as well as in LPS/IFNγ‐differentiated M1 macrophages (Fig EV3B).

These results demonstrate that ATX inhibition leads to signaling events on the downstream axis in cells that are relevant to the observed phenotypes. Crucially, the stronger effect of the Cpd17 inhibitor, specifically on RhoA activation, at least partially explains the consistent effect of the type IV inhibitor Cpd17 to ameliorate the effects in all our phenotypic assays. Our *in vitro* studies thus collectively suggested further analysis of the Cpd17 ATX inhibitor for therapeutic efficacy *in vivo*.

### Cpd17 decreases acute liver damage in CCl_4_ ‐induced liver injury *in vivo*

**Figure 6:**
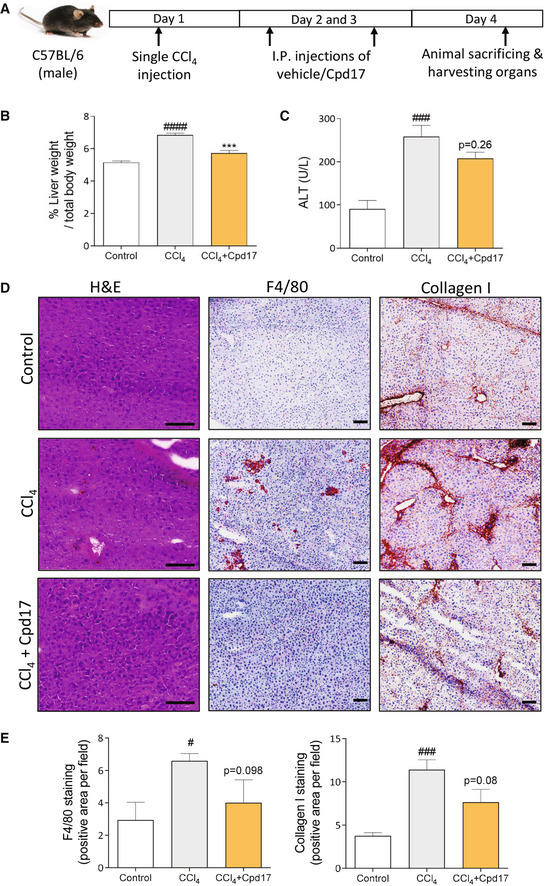
Cpd17 ameliorated CCl_4_ induced acute liver injury ASchematic showing CCl_4_‐mediated liver disease induction and Cpd17 treatment regimen.BLiver weight to total body weight ratio from control (*n* = 5), CCl_4_ (*n* = 5) and CCl_4_ + Cpd17 (*n* = 4) mice. Bars represent the Mean + SEM. Statistical analysis was performed by one‐way analysis of variance (ANOVA) with Bonferroni *post hoc* test. ^####^ *P* < 0.0001 denotes significance versus control (healthy mice); ***P < 0.001 denotes significance versus CCl_4_ mice.CSerum ALT levels from control (*n* = 5), CCl_4_ (*n* = 5) and CCl_4_ + Cpd17 (*n* = 4) mice. Bars represent the Mean + SEM. Statistical analysis was performed by one‐way analysis of variance (ANOVA) with Bonferroni *post hoc* test. ^###^ *P* < 0.001 denotes significance versus control (healthy mice); *P* = 0.26 denotes significance versus CCl_4_ mice.D, ERepresentative images (scale = 100 μm) (D) and quantitative analysis (E) of liver sections from control (*n* = 5), CCl_4_ (*n* = 5) and CCl_4_ + Cpd17 (*n* = 4) mice and stained with H&E, collagen‐I and F4/80. Bars represent the Mean + SEM. Statistical analysis was performed by one‐way analysis of variance (ANOVA) with Bonferroni *post hoc* test. ^#^ *P* < 0.05, ^###^ *P* < 0.001 denotes significance versus control (healthy mice); *P* = 0.098 or *P* = 0.08 denotes significance versus CCl_4_ mice. Source data are available online for this figure.

The carbon tetrachloride (CCl_4_)‐induced liver injury mouse model was our first choice for analyzing the *in vivo* efficacy of Cpd17 (Fig 6A). We observed that liver weights (normalized to body weight) were significantly increased in diseased mice compared with healthy control mice, and liver weights of the Cpd17‐treated mice were significantly lower compared with the diseased mice (Fig 6B). In addition, plasma transaminase (alanine transaminase, ALT) levels of mice treated with Cpd17 were also significantly reduced, compared with CCl_4_‐treated mice (Fig 6C). Histological staining of the liver tissues showed an increase in inflammation, collagen I, and F4/80 protein expression in the CCl_4_‐induced mouse model, as expected, Cpd17 notably decreased all these markers upon treatment (Fig 6D and E). Altogether, these results suggest that inhibition of ATX by Cpd17 treatment leads to reduced liver inflammation and fibrogenesis in a CCl_4_‐induced liver injury mouse model.

### Cpd17 ameliorates liver fibrogenesis and inflammation in methionine choline‐deficient (MCD)‐diet‐induced NASH mouse model

**Figure 7:**
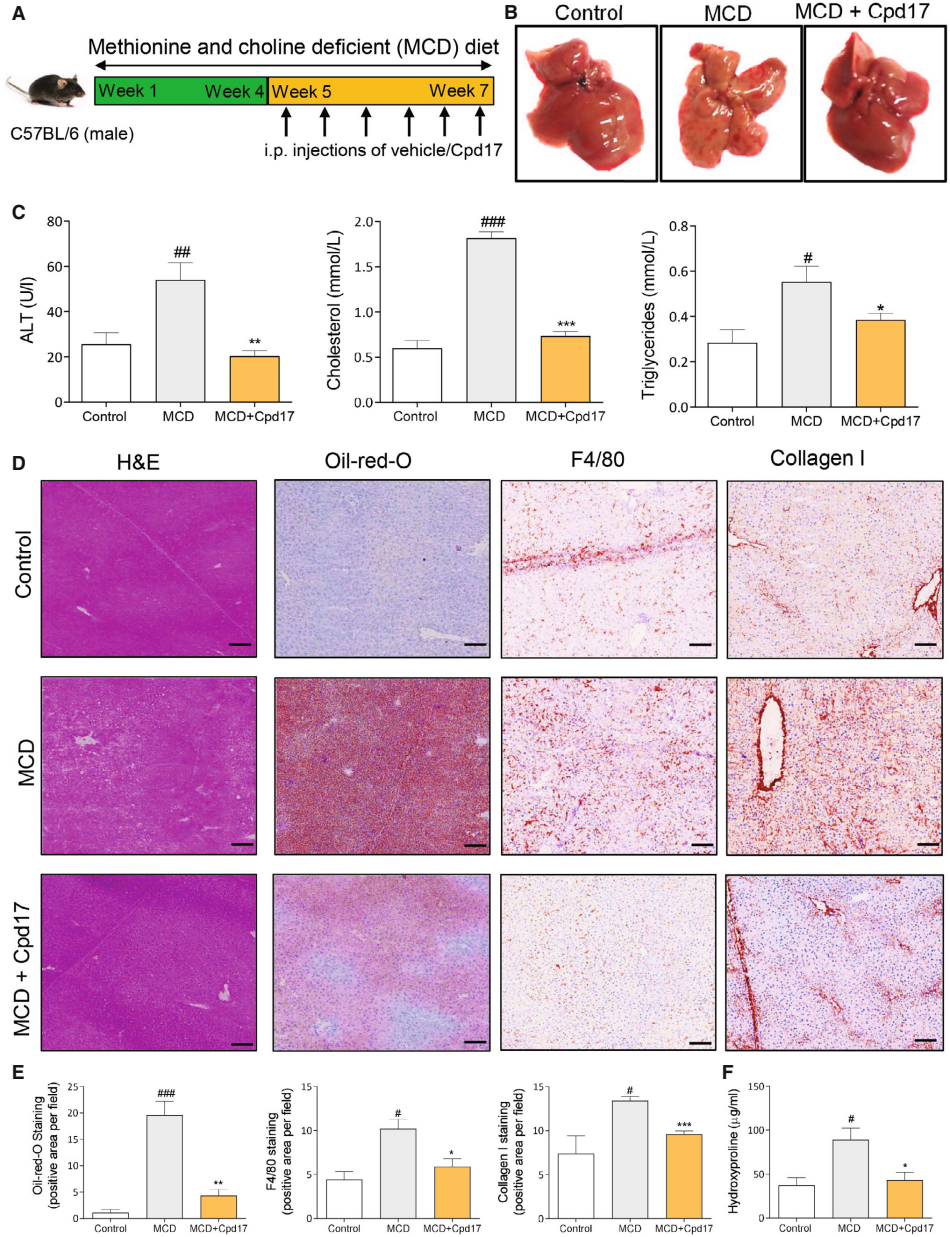
Cpd17 attenuated steatosis, inflammation and fibrosis in MCD‐diet‐induced NASH mice ASchematic showing NASH induction and Cpd17 treatment regimen.BRepresentative macroscopic images of livers from control (*n* = 5), MCD (*n* = 6) and MCD + Cpd17 (*n* = 6).CSerum ALT, serum cholesterol and serum triglyceride levels in control (*n* = 5), MCD (*n* = 6) and MCD + Cpd17 (*n* = 6) mice. Bars represent the Mean + SEM. Statistical analysis was performed by one‐way analysis of variance (ANOVA) with Bonferroni *post hoc* test. ^#^ *P* < 0.05, ^##^ *P* < 0.01, ^###^ *P* < 0.001 denotes significance versus control (healthy, chow‐diet fed mice); *P < 0.05, **P < 0.01, ***P < 0.001 denotes significance versus MCD‐diet‐fed mice.D, ERepresentative images (scale = 100 μm) (D) and quantitative analysis (E) of liver sections from control (*n* = 5), MCD (*n* = 6) and MCD + Cpd17 (*n* = 6) mice and stained with H&E, oil‐red‐O and Collagen I. Bars represent the Mean + SEM. Statistical analysis was performed by one‐way analysis of variance (ANOVA) with Bonferroni *post hoc* test. ^#^ *P* < 0.05, ^###^ *P* < 0.001 denotes significance versus control (healthy mice); *P < 0.05, **P < 0.01, ***P < 0.001 denotes significance versus MCD‐diet‐fed mice.FLiver hydroxyproline levels as assessed in livers from control (*n* = 5), MCD (*n* = 6) and MCD + Cpd17 (*n* = 6) mice. Bars represent the Mean + SEM. Statistical analysis was performed by one‐way analysis of variance (ANOVA) with Bonferroni *post hoc* test. ^#^ *P* < 0.05 denotes significance versus control (healthy mice); *P < 0.05 denotes significance versus MCD‐diet‐fed mice.

Finally, we investigated the therapeutic efficacy of Cpd17 *in vivo* in a methionine‐ and choline‐deficient (MCD)‐diet‐induced NASH mouse model (Fig 7A). Morphological examination of the liver tissues directly after sacrificing showed a distinctly visible pale and gross appearance suggesting hepatocellular damage in MCD‐diet‐induced NASH mice as compared with the healthy control group; treatment with Cpd17 improved this appearance considerably (Fig 7B). Moreover, plasma ALT levels were significantly reduced in mice treated with Cpd17 compared with MCD‐diet‐fed NASH mice, further suggesting Cpd17 significantly improved liver function (Fig 7C). Total plasma cholesterol and triglyceride levels, indicators for liver steatosis (or NAFLD), increased significantly upon MCD diet‐induced NASH were significantly decreased upon treatment with Cpd17 (Fig 7C).

Furthermore, we found that treatment with ATX inhibitor Cpd17 ameliorated hepatic inflammation and steatosis, as examined by H&E and Oil‐red‐O staining, respectively (Fig 7D and E). In addition, the MCD‐diet feeding resulted in increased intrahepatic protein expression of F4/80 (macrophage marker) and collagen I, compared with controls, as examined by immunostaining (Fig 7D and E). Strikingly, *in vivo* treatment with our ATX inhibitor led to a significantly decreased protein expression of both collagen I and F4/80, as shown in the microscopic images and quantitative staining analysis.

Moreover, total collagen content in the livers was evaluated through hydroxyproline assay that further confirmed an increased total collagen I concentration in MCD‐livers, which was decreased in Cpd17‐treated mice (Fig 7F).

**Figure EV4:**
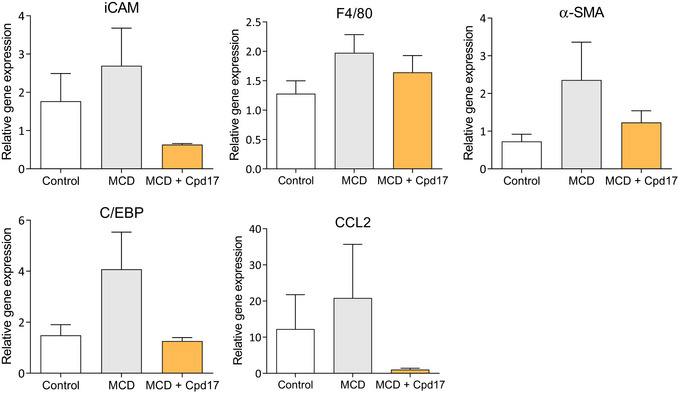
Effect of Cpd17 on NASH related genes in MCD‐mouse model Relative F4/80, CCL2, iCAM, α‐SMA and C/EBP gene expression levels (normalized with GAPDH) as analyzed in control (*n* = 5), MCD (*n* = 6) and MCD + Cpd17 (*n* = 6) treated mouse livers. Bars represent the Mean + SEM.

In addition, quantitative PCR revealed a decrease upon Cpd17 treatment in inflammatory markers F4/80, CCL2 and intracellular cell adhesion molecule (iCAM1), HSC‐specific marker α‐SMA and liver steatosis marker CCAAT‐enhancer‐binding proteins (C/EBP) (Rahman *et al*, 2007) (Fig EV4).

### Cpd17 differentially regulate downstream signaling *in vivo*

**Figure EV5:**
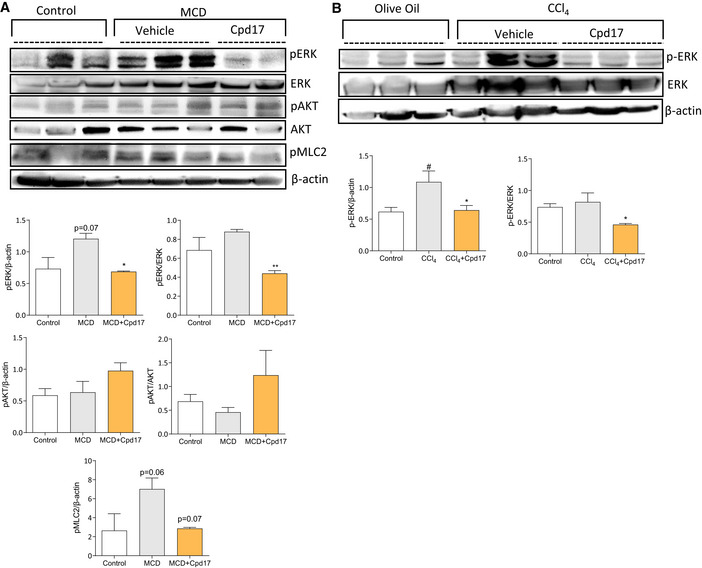
Cpd17 differentially regulate downstream signalling *in vivo* in MCD‐diet‐induced NASH mouse model and in CCl_4_‐induced liver injury mouse model ARepresentative Western‐blot images and quantitation of pERK normalized to ERK or β‐actin as analyzed in control (*n* = 5), CCl_4_ (*n* = 5) and CCl_4_ + Cpd17 (*n* = 4) mouse livers. Bars represent the Mean + SEM; Statistical analysis was performed by one‐way analysis of variance (ANOVA) with Bonferroni *post‐hoc* test; ^#^ *P* < 0.05 denotes significance versus control (healthy mice); *P < 0.05 denotes significance versus CCl_4_ mice.BRepresentative Western‐blot images and quantitation of pERK, pAKT, pMLC2 normalized to ERK, AKT or β‐actin as analyzed in control (*n* = 5), MCD (*n* = 6) and MCD + Cpd17 (*n* = 6) mouse livers. Bars represent the Mean + SEM. Statistical analysis was performed by one‐way analysis of variance (ANOVA) with Bonferroni *post‐hoc* test. *P* = 0.07, *P* = 0.06 denotes significance versus control (healthy mice); *P < 0.05, **P < 0.01, *P* = 0.07 denotes significance versus MCD‐diet fed mice. Source data are available online for this figure.

Next, we investigated the mechanism of Cpd17 *in vivo* in both MCD‐diet‐ and CCl_4_‐induced liver disease models. In line with our *in vitro* analysis, we analyzed ERK, AKT, and RhoA signaling pathways. It is important to note that these signaling pathways are involved in several cellular processes independent of ATX signaling, and analysis of the *in vivo* results can be challenging. Cpd17 significantly decreased ERK activation in both animal models (Fig EV5), consistent with the *in vitro* results (Fig 5C), but did not result in significant changes in pAKT activation *in vivo* (Fig EV5). Finally, the levels of phospho‐Myosin Light Chain 2 (pMLC2) to monitor RhoA pathway activation showed an increase in MCD mice, which was reduced to control levels following Cdp17 treatment; however, both changes were marginally not significant (Fig EV5B). These results confirm that Cpd17 affects *in vivo* the same disease‐relevant pathways as it affects *in vitro* and its mode of action.

## Discussion

The ATX/LPA signaling axis has previously shown to be involved with the initiation and progression of liver diseases (Nakagawa *et al*, 2011; Kondo *et al*, 2014; Yamazaki *et al*, 2017; Ando *et al*, 2018; Fujimori *et al*, 2018; Joshita *et al*, 2018). Here, we show significant upregulation of ATX gene and protein expression in human liver NASH and cirrhosis patients, which we recapitulated in CCl_4_‐induced liver injury and MCD diet‐induced NASH mouse models. Having confirmed and strengthened the connection between ATX and liver disease, we evaluated the therapeutic efficacy of Cpd17 in different cell types using different read‐out parameters and *in vitro* assays. Initial studies were performed with different concentrations (100 nM to 5 μM) of Cpd17 based on which optimal concentration of 1 μM was selected for further *in vitro* studies which also aligned with previous reports where other ATX inhibitors have been tested *in vitro* (Castelino *et al*, 2016; Ho *et al*, 2020). We showed that a (yet unexplored) type IV ATX inhibitor Cpd17 at 1 μM concentration, compared with previously reported type I ATX inhibitor PF8380, possess stronger inhibitory effects in a variety of *in vitro* assays in hepatocytes, pro‐inflammatory macrophages and activated HSCs. We found that ATX inhibition by Cpd17 attenuated TGFβ‐induced expression of fibrotic markers in human HSCs, and LPS/IFNγ‐stimulated expression of inflammatory markers in both mouse and human macrophages. We also showed a decrease in collagen I contraction of HSCs upon ATX inhibition, a measure of functional cellular activation (Corin & Gibson, 2010), and decreased migration of activated HSCs and macrophages. LPA via Gα(12/13) activates RhoA kinase pathway by which it regulates HSCs morphology by organizing the actin cytoskeleton, thereby HSCs‐ECM interaction and HSCs contraction thus fibrotic phenotypes (Yanase *et al*, 2000). While examining the effect of Cpd17 and PF8380 on ATX/LPA‐related downstream signaling, we evidenced that they notably affected the RhoA pathway both *in vitro* and *in vivo*. We also showed broader effects through the MAPK/ERK and AKT/PKB pathways, and that these signals go through LPA receptors. We thus establish that the mechanisms underlying the ATX inhibition, using a type IV inhibitor, have an effect on downstream signaling processes that influence inflammation and fibrogenesis.

The cell‐based assays clearly demonstrated that Cpd17 has a notably better potential in ameliorating fibrotic phenotypes and downstream signaling responses, compared with PF8380, which had already shown effectiveness in attenuating CCl_4_‐induced liver cirrhosis (Kaffe *et al*, 2017). PF8380 is a so‐called type I inhibitor of ATX, which affects LPC hydrolysis to the signaling LPA molecule. However, the type I inhibitor PF8380 does not occupy the allosteric tunnel of ATX that leads to activity modulation (Salgado‐Polo *et al*, 2018) and has been suggested to be implicated in presenting LPA to its cognate GPCRs (Nishimasu *et al*, 2011). In contrast, Cpd17 (Keune *et al*, 2017) is a type IV inhibitor that prevents substrate binding and also occupies the ATX tunnel. Cpd17 is structurally (but not chemically) related to GLPG1690 (Salgado‐Polo & Perrakis, 2019), which has shown promise in treating IPF (Desroy *et al*, 2017).

Based on our *in vitro* results, we further evaluated ATX inhibition by Cpd17 *in vivo* in acute liver injury and NASH mouse models. We used the CCl_4_‐induced liver fibrosis model (Scholten *et al*, 2015) and the MCD‐diet‐induced NASH mouse model (Machado *et al*, 2015) which showed characteristic hepatocytes damage and/or lipid accumulation, accompanied by intrahepatic inflammation and fibrosis, as reported previously (Nevzorova *et al*, 2020). Cpd17 has an effect both on MCD diet‐induced hepatic steatosis, and on the CCl_4_‐induced liver injury model. These results are in accordance with the previous study in which adipose‐specific ATX deficiency exhibited reduced lipid accumulation and hepatic steatosis associated with high‐fat diet, while ATX overexpression was found to aggravate steatosis (Brandon *et al*, 2019). Interestingly, previous studies with the potent Ex_31 inhibitor had no effect on CCl_4_‐induced liver fibrosis and choline‐deficient amino acid‐defined diet‐induced liver injury (Baader *et al*, 2018). PAT‐505, a type III ATX inhibitor, showed reduced fibrosis development in a high‐fat diet‐induced NASH mouse model with no significant effect on steatosis, inflammation, or hepatocyte ballooning (Bain *et al*, 2017). Moreover, inhibition by PF8380 in CCl_4_‐induced liver disease models has shown to reduce plasma ATX activity and liver LPA levels by approximately 50%, and attenuate fibrosis‐based on histopathological scoring and collagen deposition in the liver (Kaffe *et al*, 2017). Based on our results, ATX inhibition by Cpd17 showed protection against CCl_4_‐induced acute liver injury, and decreased steatosis, inflammation, and fibrosis in the NASH mouse model.

The analysis of the signaling pathways examined *in vitro* were largely consistent with *in vivo*. Cdp17 significantly decreased pERK signaling in both animal models, and also decreased the pMLC marker of RhoA signaling back to physiological levels, albeit the latter change was marginally not statistically significant (*P* = 0.07). Curiously, pAKT levels showed contradicting results upon Cpd17 treatment *in vitro* (decrease) and *in vivo* (increase). However, pAKT/AKT levels are differentially regulated in different cell types during liver injury and regeneration. On one hand, AKT activation improves hepatic regeneration and is involved in restricting pro‐inflammatory and promoting anti‐inflammatory responses via negative regulation of TLR and NF‐κB signaling in macrophages (Vergadi *et al*, 2017); on the other hand, PI3K/AKT pathway blockade in HSCs has been shown to suppress fibrotic responses by inhibiting HSCs proliferation and collagen synthesis (Son *et al*, 2013). Interestingly, the ERK‐AKT axis has been suggested to act as a switch regulating liver regeneration or fibrosis via liver sinusoidal endothelial cells (LSECs)‐HSCs communication (Lao *et al*, 2018). Thus, the expected effect of Cpd17 through LPA‐mediated signaling is likely masked by the effect of other relevant regulatory pathways in the MAP/AKT signaling pathway which affect differentially different cell types in the liver.

The excellent *in vitro* and *in vivo* efficacy of Cpd17 in liver disease models, together with GLPG1690 efficacy in treating IPF, argue that Type IV compounds are very promising agents in treating fibrotic diseases and possibly other pathologies related to the LPA/ATX axis. It must be noted, though, that the phase III Isabella trial for GLPG1690 was terminated due to adverse effects. Detailed results from the Isabella trial have not been published to this moment, and it is thus hard to speculate why it failed. If that was due to the off‐target effects of the specific compound, this suggests a need to assess alternative chemical scaffolds such as Cpd17.

Based on the results in this study, we have clearly demonstrated the role of the ATX‐LPA axis in NASH and liver cirrhosis. We have also shown that inhibition of ATX, using a type IV inhibitor Cpd17, ameliorates fibrosis, inflammation, and steatosis in HSCs and macrophages *in vitro*, as well as in NASH and liver cirrhosis mouse models. We also note, that while Cpd17 clearly effects phenotypes relevant to fibrosis in liver cells, it cannot be excluded that its therapeutic potential *in vivo*, might be related to an interplay with the immune system, and specifically with the infiltration of T cells, as recently shown in a tumor model (preprint: Matas‐Rico *et al*, 2021). Regardless, type IV ATX inhibitors have clear promise in nonalcoholic steatohepatitis and liver cirrhosis. Furthermore, our results also suggest it worth exploring the role of the ATX/LPA axis and the effect of its inhibition in models of liver cancer, with potential therapeutic opportunities for HCC.

## Materials and Methods

### Transcriptomic analysis

For ATX (ENPP2) analysis in human tissues, the transcriptomic datasets of liver tissue from patients with liver cirrhosis (GSE14323) (Mas *et al*, 2009), and nonalcoholic steatohepatitis (GSE63067) (Frades *et al*, 2015), from the National Center of Biotechnology Information Gene Expression Omnibus database (NCBI‐GEO) were analyzed using GEO2R.

### Human liver specimens

Human liver specimens were obtained from the autopsy of patients with NASH (*n* = 5) and cirrhosis (*n* = 5) anonymously provided by the Laboratory Pathology Netherlands (LabPON). Normal liver tissue (*n* = 4) was collected from patients receiving hepatic resections for nontumoral diseases, including hepatic adenoma and focal nodular hyperplasia. Upon Institutional Review Board approval and after written informed consent from patients, the tissue specimens were collected. The use of human tissues was approved by the Local Medical Ethics Committee, and the experimental protocols were performed in accordance with institutional guidelines and regulations. The experiments conformed to the principles set out in the WMA Declaration of Helsinki and the Department of Health and Human Services Belmont Report.

### AKT, ERK, and MLC2 phosphorylation by Western blotting in liver tissues

For Western blotting, liver tissues were homogenized using an electric homogenizer in cold RIPA buffer (5 mg/300 μl), supplemented with protease inhibitors (Pierce), phospho‐stop (Roche) on ice. The homogenized tissues were maintained at constant agitation for 1 h at 4°C, and centrifuged for 20 min at 12,000 rpm/4°C. The clear supernatants were transferred to a fresh tube, and protein concentration was measured using a BCA protein assay kit (Pierce). LDS sample buffer (NuPAGE, Invitrogen) and 1 mM dithiothreitol (DTT) were added to the lysate. 20 μg total protein was loaded on SDS‐PAGE precast gradient gels (4–12% Nu‐Page Bis‐Tris, Invitrogen), followed by transfer to nitrocellulose membrane. Nonspecific protein binding was blocked by 5% BSA in PBS‐Tween (0.1%); primary antibodies (Appendix Table S1). They were incubated overnight at 4°C in PBS‐tween with 5% BSA, containing 0.1% NaN_3_. Blots were then incubated for 1 h at RT with monoclonal anti‐β‐actin antibody prepared in PBS‐tween with 5% skimmed milk containing 0.1% NaN_3_. Horseradish peroxidase‐conjugated secondary antibodies (Appendix Table S1) were incubated for 1 h at room temperature in PBS‐tween with 2.5% BSA and developed using ECL Western blot reagent (Thermo Scientific).

### Cell lines

Human hepatic stellate cells (LX2 cells), provided by Prof. Scott Friedman (Mount Sinai Hospital, New York, NY, USA), were cultured in DMEM‐Glutamax medium (Invitrogen, Carlsbad, CA, USA) supplemented with 10% fetal bovine serum (FBS, Lonza, Verviers, Belgium) and antibiotics (50 U/ml Penicillin and 50 μg/ml streptomycin, Sigma, St. Louis, MO, USA). Murine RAW 264.7 macrophages and human THP1 monocytes, obtained from the American Type Culture Collection (ATCC, Manassas, VA, USA), were cultured in Roswell Park Memorial Institute (RPMI) 1640 medium (Lonza) supplemented with 10% FBS (Lonza), 2 mM L‐glutamine (Sigma) and antibiotics. Human HepG2 cells (ATCC) were cultured in DMEM‐high glucose medium (Lonza) supplemented with 10% FBS and antibiotics. All the cell lines used in this study were authenticated with STR profiling and were tested regularly for the absence of mycoplasma contamination by PCR.

### AKT and ERK phosphorylation by Western blotting

100,000 LX2 or 20,000 RAW cells were seeded in 6‐ and 12‐well tissue culture plates and allowed to grow for 24 h in DMEM (GIBCO, Life Technologies) containing 10% FBS and antibiotics, containing 5 ng/ml TGFβ (for LX2 cells), or 100 ng/ml LPS and 10 ng/ml IFNγ (for RAW cells). Next, they were washed twice with PBS and serum starved overnight. Mixes with 1 μM 18:1 LPC, 1 μM 18:1 LPA, 20 nM ATX, 1 μM PF8380, 1 μM Cpd17, or 1 μM Ki16425 were incubated for 30 min in serum‐free medium containing 0.05% (w/v) fatty acid‐free BSA (total volume 1 ml). Medium from the plates was removed and replaced with 1 ml of ATX‐inhibitor mixture. Cells were stimulated for 10 min, medium was removed, and plates were immediately frozen on dry ice and stored at −80°C. For Western blotting, cells were lysed in RIPA buffer, supplemented with protease inhibitors (Pierce), 20 mM NaF and 1 mM Orthovanadate, and spun down. Protein concentration was measured using a BCA protein assay kit (Pierce). LDS sample buffer (NuPAGE, Invitrogen) and 1 mM dithiothreitol (DTT) were added to the lysate. 20 μg total protein was loaded on SDS–PAGE precast gradient gels (4–12% Nu‐Page Bis‐Tris, Invitrogen), followed by transfer to nitrocellulose membrane. Nonspecific protein binding was blocked by 5% BSA in PBS‐Tween (0.1%); primary antibodies (Appendix Table S1). They were incubated overnight at 4°C in PBS‐tween with 5% BSA, containing 0.1% NaN_3_. Blots were then incubated for 1 h at RT with monoclonal anti‐β‐actin antibody prepared in PBS‐tween with 5% skimmed milk containing 0.1% NaN_3_. Horseradish peroxidase‐conjugated secondary antibodies (Appendix Table S1) were incubated for 1 h at room temperature in PBS‐tween with 2.5% BSA and developed using ECL Western blot reagent (Thermo Scientific).

### Rho GTPase biosensor

A FRET pair consisting of RhoA‐Cerulean3 and PKN fused to circularly permuted Venus was used (Kedziora *et al*, 2016). The CRIB domain of PAK and HR1 region of PKN were used as the effector domain for activated Rac1/Cdc42 and RhoA, respectively. Experiments were performed in HEPES‐buffered saline (containing 140 mM NaCl, 5 mM KCl, 1 mM MgCl_2_, 1 mM CaCl_2_, 10 mM glucose, 10 mM HEPES), pH 7.2, at 37°C. Cells were allowed to adhere overnight on uncoated coverslips, after which they were serum‐starved and transfected with the indicated biosensor for 24 h. Next, the coverslips were placed on a thermostatted (37°C) inverted Nikon Diaphot microscope and excited at 425 nm. Donor and acceptor emission were detected simultaneously with two photomultipliers, using a 505 nm beam splitter and optical filters: 470 ± 20 nm (CFP channel) and 530 ± 25 nm (YFP channel). The emission data were analyzed using the Fiji software and normalized to control cells. At least three independent experiments were analyzed for every condition (10 fields of view/condition, 3–5 cells/field of view, >30 cells/condition). FRET was expressed as the ratio between acceptor and donor signals, set at 1 at the onset of the experiment.

### Surface biotin labeling

For LPA1‐HA internalization, LX2 cells were grown on 6‐well plates 24 h, medium was overnight in serum‐free DMEM medium, LPA1‐HA was overexpressed in LX2 cells upon transfection with Fugene6 at a 1:3 (μg/ml) ratio overnight. Between each of the previous steps, cells were washed twice with PBS. For LPA1‐HA internalization, cells were stimulated with the indicated reagents serum‐free DMEM containing 0.1% fatty acid‐free BSA for 15 min. Next, the cells were transferred to ice, washed in ice‐cold PBS, and surface labeled with 0.2 mg/ml Sulfo‐NHS‐SS‐Biotin (Thermo Scientific) for 30 min. Biotin‐labeled LPA1‐HA was detected using streptavidin beads (Pierce) and conjugated anti‐HA antibody (3F10 from Roche Diagnostics; 1:1000).

### Effect of ATX inhibitors on lipid biogenesis in human hepatocytes (HepG2 cells)

HepG2 cells were treated with palmitate (0.2 mM) in complete culture medium, supplemented with 1% BSA, with and without PF8380 or Cpd17 (1 μM) for 48 h. Thereafter, cells were washed twice with PBS, and either lysed with RNA lysis buffer or fixed and stained using an Oil‐red‐O staining kit (Sigma) as per the manufacturer's instructions.

### Effects of ATX inhibitors on macrophages

Mouse RAW macrophages or human THP1 monocytes (with 50 ng/ml phorbol 12‐myristate 13‐acetate, PMA, Sigma) were plated in 12‐well plates (5 × 10^5^ cells/ml) and incubated overnight. They were then incubated with cell medium alone, M1 stimulus (100 ng/ml LPS and 10 ng/ml IFNγ) and M2 stimulus (10 ng/ml IL‐13 and 10 ng/ml IL‐4), with or without 1 μM of PF8380 or Cpd17 for 24 h. For wound healing analyses, a 200 μl pipet was used to make a scratch on the surface of the cells. Pictures were taken at 0 h and 24 h after incubation with transmitted light microscopy. Afterward, cells were lysed with an RNA lysis buffer for quantitative real‐time PCR analyses.

### The effect of ATX inhibitor Cpd17 on human LX2 cells

Cells were seeded in 12‐well plates (1 × 10^5^ cells/ml) or 24‐well plates (6 × 10^4^ cells/ml) and cultured overnight. To study the effect of the inhibitor, cells were starved with serum‐free medium overnight, and then treated with medium alone or 5 ng/ml human recombinant TGFβ with or without 1 μM PF8380 or Cpd17. A 200 μl pipet tip was used to create a scratch in the cell monolayer (12‐well plates). Images were taken at 0 h and 24 h after the scratch using transmitted light microscopy (Evos microscope). The percentage of cells migrated into the wound was measured by the difference in scratch diameter between 0 and 24 h, normalized to untreated (control) cells. Thereafter, cells were lysed with RNA lysis buffer to perform quantitative real‐time PCR analysis. In addition, cells (24‐well plates) were fixed with acetone: methanol (1:1) solution, dried, and stained for collagen I and α‐SMA.

### 3D‐collagen I contraction assay

3D‐collagen I gel contraction assay has been done as described earlier (Akcora *et al*, 2018). Briefly, a 5.0 ml collagen suspension with 3.0 ml collagen G1 (5 mg/ml, Matrix biosciences, Morlenbach, Germany), 0.5 ml 10× M199 medium (Sigma), 85 μl 1N NaOH (Sigma), and 1.415 ml of sterile water was prepared. This suspension was mixed with 1 ml (2 × 10^6^) LX2 cells. 0.6 ml of the collagen‐gel suspension was plated in a 24‐well plates and incubated at 37°C/5% CO_2_ for 1 h. Polymerized gels were then incubated with serum‐free medium with or without TGFβ (5 ng/ml) together with 1 μM of PF8380 or Cpd17. Thereupon, gels were detached from the bottom of the culture wells. Pictures were taken with a digital camera at 0 h and 24 h. The area of the gel was digitally measured and normalized to the area of the inner size of the well in each image.

### Animal experiments

All the animal experiments performed in this study were in accordance with the guidelines and regulations for the Care and Use of Laboratory Animals, Utrecht University, the Netherlands. The protocols were approved by the Institutional Animal Ethics Committee of the University of Twente, the Netherlands. Eight‐week‐old male C57BL/6NRj mice purchased from Janvier Inc. Labs (Le Genest, St. Isle, France) were housed in standard SPF conditions in a Tecniplast IVC system (cage type 2 L, blue line).

Carbon tetrachloride (CCl_4_)‐induced acute liver injury model: Male C57BL/6NRj mice were treated with a single intraperitoneal injection of olive oil (Sigma) or CCl_4_ (Sigma, 0.5 ml/kg in olive oil) at day 1. At day 2 and day 3, CCl_4_‐treated mice received intraperitoneal administration of 5 mg/kg Cpd17 or vehicle treatment (*n* = 5 per group). On day 4, all mice were sacrificed, blood and livers were collected for subsequent analysis.

CCl_4_‐induced liver cirrhosis mouse model: Male balb/c mice (20–22 g) were treated with olive oil (*n* = 5) or increasing doses of CCl_4_ (week 1: 0.5 ml/kg; week 2: 0.8 ml/kg and week 3–8: 1 ml/kg prepared in olive oil, *n* = 8) twice weekly by intraperitoneal injections for 8 weeks as described previously (Bansal *et al*, 2011). All mice were sacrificed at week 8, and livers were collected for subsequent analysis.

For the methionine and choline‐deficient (MCD) diet‐induced NASH model, C57BL/6NRj mice were fed with a standard chow or MCD diet for 7 weeks. Starting at week 5, the mice received intraperitoneal administrations of 5 mg/kg Cpd17 or vehicle treatment 2 times per week, for a duration of 3 weeks, *n* = 5–6 per group. All mice were sacrificed 24 h after the last treatment, blood and livers were collected for further analysis. Alanine aminotransferase (ALT), aspartate aminotransferase (AST), total plasma cholesterol levels, and plasma triglycerides levels were measured by standard automated laboratory methods.

### Hydroxyproline assay

Total liver hydroxyproline concentration was determined using a hydroxyproline assay (Sigma), a commonly used method for quantifying total collagen content, according to the manufacturer's instructions. In short, 10 mg liver tissue was homogenized in 100 μl MilliQ, with a mechanical homogenizer (Ultra‐Turrax, IKA), and transferred to a pressure‐tight polypropylene vial with PTFE‐lined cap. 100 μl concentrated hydrochloric acid (12 M) was added, the vials capped tightly, and samples were hydrolyzed at 95°C for 24 h. Thereafter, samples were centrifuged at 10,000 *g* for 3 min. 50 μl of supernatant was transferred to a 96‐well plates, which was allowed to dry at 60°C overnight. To each well, 100 μl of Chloramine T/Oxidation buffer mixture was added and incubated for 5 min. Thereafter, 100 μl of p‐Dimethylaminobenzaldehyde (DMAB; Ehrlich's Reagent) diluted with perchloric acid/isopropanol solution was added to each well and incubated for 90 min at 60°C. Absorbance was measured at 560 nm and results quantified using known standard.

### Immunohistochemistry

Liver tissues were harvested and transferred to Tissue‐Tek optimum‐cutting temperature (O.C.T) embedding medium (Sakura Finetek, Torrance, CA, USA), and snap‐frozen in 1‐methyl butane chilled on dry ice. Cryosections (7 μm) were cut using a Leica CM 3050 cryostat (Leica Microsystems, Nussloch, Germany). The sections were air‐dried and fixed with acetone for 20 min. Cells or tissue cryosections were rehydrated with PBS and incubated with the primary antibody (Appendix Table S2) for 1 h at room temperature or at 4°C overnight. Formalin‐fixed paraffin embedded (FFPE) sections were deparaffinized in xylene, and rehydrated in graded ethanol, and distilled water. Antigen retrieval was achieved by overnight incubation at 60°C in 0.1 M Tris/HCl buffer (pH 9.0) followed by the overnight incubation with the primary antibody. For tissue sections, endogenous peroxidase activity was blocked by 3% H_2_O_2_ prepared in methanol for 30 min. Cells or sections were then incubated with horseradish peroxidase (HRP)‐conjugated secondary antibody for 1 h at room temperature and thereafter incubated with HRP‐conjugated tertiary antibody for 1 h at room temperature. Thereafter, peroxidase activity was developed either using AEC (3‐amino‐9ethylcarbazole) substrate kit (Life Technologies, Carlsbad, CA, USA) for the cryosections or DAB (3,3′‐Diaminobenzidine) substrate kit (Pierce, Thermo Scientific) for the FFPE sections for 20 min, and nuclei were counterstained with hematoxylin (Sigma). Cells or sections were mounted in Aquatex mounting medium (Merck, Sigma) for cryosections or VectaMount (VectorLabs) for FFPE sections following dehydration in graded ethanol. The staining was visualized, and images were captured using light microscopy (Nikon eclipse E600 microscope, Nikon, Tokyo, Japan). Furthermore, sections were scanned using Hamamatsu NanoZoomer Digital slide scanner 2.0HT (Hamamatsu Photonics, Bridgewater, NJ, USA).

### Immunofluorescence

Cells were fixed with acetone: methanol (1:1) and air‐dried for 20 min. The cells were rehydrated with cold PBS and incubated with collagen I primary antibody (1:100) for 1 h at room temperature. Cells were then incubated with Alexa‐488‐labeled secondary antibody (1:100) for 1 h at room temperature. Thereafter, the cells were fixed with DAPI‐containing antifade mounting medium (Merck, Sigma). The staining was visualized, and images were captured using fluorescence microscopy (Evos).

### Oil‐Red‐O staining

Oil‐Red‐O stock solution was prepared by dissolving 0.3 g Oil‐Red‐O (Sigma) in 100 ml of isopropanol. Cells or sections were fixed in 4% formalin for 20 min and then stained with Oil‐Red‐O as per the manufacturer's instructions. Briefly, formalin‐fixed cells or sections were rinsed with 60% isopropanol followed by staining with freshly prepared Oil‐Red‐O working solution for 15 min. Thereafter, cells or sections were rinsed with 60% isopropanol and nuclei were counterstained with hematoxylin (Fluka Chemie, Sigma). Finally, cells or sections were washed with tap water and mounted with Aquatex mounting medium (Merck, Sigma).

### Hematoxylin and eosin staining

Sections were fixed with 4% formalin for 20 min and then rinsed with distilled water. The sections were incubated with hematoxylin for 15 min followed by washings with tap water. Thereafter, sections were incubated with eosin solution for 1.5 min followed by washing in 96% ethanol, dehydration with ethanol and were mounted with VectaMount mounting medium (Vector Laboratories, Burlingame, CA).

### Quantitative histological analysis

For quantitative histological analysis, high‐resolution scans were viewed using NanoZoomer Digital Pathology (NDP2.0) viewer software (Hamamatsu Photonics). About 20 images (100×) of each entire section (from NDP) were imported into ImageJ and were analyzed quantitatively at a fixed threshold. All the primary antibodies used in this study have been pretested for specificity. The staining performed in the study included the negative control (without primary antibody) to confirm the specificity of the staining and showed no nonspecific staining.

### RNA extraction, reverse transcription, and quantitative real‐time PCR

Total RNA from cells and liver tissues was isolated using GenElute Total RNA Miniprep Kit (Sigma) and SV total RNA isolation system (Promega Corporation, Madison, WI, USA), respectively, according to the manufacturer's instructions. The RNA concentration was quantified with a UV spectrophotometer (Nanodrop Technologies, Wilmington, DE, USA). Total RNA (1 μg) was reverse transcribed using the iScript cDNA Synthesis Kit (Bio‐Rad, Hercules, CA, USA). All primers were purchased from Sigma‐Genosys (Haverhill, UK). Real‐time PCR was performed using 2× SensiMix SYBR and Fluorescein Kit (Bioline, QT615‐05, Luckenwalde, Germany), 20 ng cDNA and pretested gene‐specific primer sets (listed in Appendix Table S3). The cycling conditions for the Bio‐Rad CFX384 Real‐Time PCR detection system was 95°C for 10 min, 40 cycles of 95°C/15 s, 72°C/15 s, and 58°C/15 s. Finally, cycle threshold (Ct) values were normalized to reference gene GAPDH or 18S and fold changes in expression were calculated using the 2^−ΔΔCt^ method.

### Study design and statistics of animal experiments

No statistical methods were used to predetermine sample size. At least five animals were used per group in all animal experiments. Animals were allocated into groups randomly, and the handling, harvest, and analysis of animals were blind to reduce subjective bias. No inclusion or exclusion criteria were conducted on the animals.

### Statistical analysis

All data are presented as the mean ± standard error of the mean (SEM). The graphs and statistical analyses were performed using GraphPad Prism version 9.1.2 (GraphPad Prism Software, Inc., La Jolla, CA, USA). Comparisons with control groups were analyzed using unpaired Student's *t*‐test and/or multiple comparisons between different groups were performed by one‐way analysis of variance (ANOVA) with Dunnett's multiple comparison or Bonferroni *post hoc* test. The differences were considered statistically significant as follows: *P < 0.05, **P < 0.01 and ***P < 0.0001. All experiments were performed at least three times independently.

## Author contributions

**Richell Booijink:** Data curation; validation; investigation; visualization; methodology; writing – original draft; writing – review and editing. **Fernando Salgado‐Polo:** Data curation; validation; investigation; visualization; methodology; writing – original draft; writing – review and editing. **Craig Jamieson:** Resources; writing – review and editing. **Anastassis Perrakis:** Conceptualization; supervision; funding acquisition; visualization; writing – original draft; project administration; writing – review and editing. **Ruchi Bansal:** Conceptualization; supervision; funding acquisition; visualization; writing – original draft; project administration; writing – review and editing.

## Disclosure and competing interests statement

The authors declare that they have no conflict of interest.

**The paper explained**

**Problem**

Liver diseases affect millions of people worldwide, causing approximately 2 million deaths every year. Among liver diseases, drug‐induced acute liver injury, nonalcoholic fatty liver disease and its severe form nonalcoholic steatohepatitis have been recognized as a major public health concern in Western countries. Currently, there is no effective treatment available for the liver diseases.

**Results**

In this paper, we targeted a lysophosphatidic acid–autotaxin pathway, known to be involved in liver injury, by using a novel type IV autotaxin inhibitor, Cpd17. We demonstrated the effectivity of Cpd17 in ameliorating acute and chronic liver injury in two mouse models. Moreover, we compared Cpd17 with a classic type I autotaxin inhibitor, PF8380, and observed that Cpd17 was more effective at inhibiting the activation of specific cells that are involved in initiation and progression of liver diseases.

**Impact**

Autotaxin inhibition, particularly by a type IV inhibitor such as Cpd17, has an excellent potential for the treatment of liver diseases.

## Supporting information



Appendix

Expanded View Figures PDF

Source Data for Expanded View

Source Data for Figure 1

Source Data for Figure 3

Source Data for Figure 4

Source Data for Figure 5

Source Data for Figure 6

## Data Availability

This study includes no data deposited in external repositories.
