# Supplementary material for: A type IV Autotaxin inhibitor ameliorates acute liver injury and nonalcoholic steatohepatitis
Source: EMBO Mol Med. 2022 Jul 14;14(9):e16333. doi: 10.15252/emmm.202216333 (PMC9449594; doi:10.15252/emmm.202216333)
Supplement: Supplementary file 1 — Appendix [file EMMM-14-e16333-s001.pdf]

APPENDIX

Table of Content

**Appendix Tables**	
**Appendix Table S1**	**Antibodies used for western blotting**
**Appendix Table S2**	**Antibodies used for the immunostainings**
**Appendix Table S3**	**Sequence of the primers used for quantitative real-time PCR**
**AKT, ERK and MLC2 phosphorylation by Western blotting in liver tissues.**	

Appendix Table S1: Antibodies used for western blotting

**Primary antibody**	**Source**	**Dilution**
phospho-Akt (Ser473) #9271	Cell Signaling Technology	1:1000
AKT #9272	Cell Signaling Technology	1:1000
Phospho-p44/42 MAPK (p-Erk1/2) (Thr202/Tyr204) #9101	Cell Signaling Technology	1:2000
p44/42 MAPK (Erk1/2) #9102	Cell Signaling Technology	1:2000
Phospho-Myosin light chain 2 (Thr18/Ser19) #3674	Cell Signaling Technology	1:1000
monoclonal anti-β-actin (clone AC-15)	Sigma	1:10000
**Secondary antibody**	**Source**	**Dilution**
Goat anti-mouse	Bio-Rad	1:5,000
Goat anti-rabbit	Pierce	1:5,000

Appendix Table S2: Antibodies used for the immunostainings

**Primary antibody**	**Source**	**Dilution**
Polyclonal goat anti-Collagen I	Southern Biotech	1:100
Rat anti-mouse F4/80	Bio-Rad	1:100
Anti-ENPP2 antibody produced in rabbit	Sigma	1:100
**Secondary antibody**	**Source**	**Dilution**
HRP-conjugated goat anti-rabbit IgG	DAKO	1:100
HRP-conjugated rabbit anti-goat IgG	DAKO	1:100
HRP-conjugated goat anti-rat IgG	DAKO	1:100
Donkey anti-rabbit Alexa Fluor 488	Thermo Fisher Scientific	1:200

Appendix Table S3: Sequence of the primers used for quantitative real-time PCR

Gene	Forward primer sequence	Reverse primer sequence	Accession no.
**Mouse Primers**			
ENPP2	AGAGGAAGTCAGCAGACCCA	ACCTTATCATCACAGGTGCAG	NM_001136077.3
CCl2	GTGCTGACCCCAAGAAGGAA	GTGCTGAAGACCTTAGGGCA	NM_011333.3
iNOS	GGTGAAGGGACTGAGCTGTT	GCTACTCCGTGGAGTGAACAA	NM_010927.4
TNFα	AGGCTGCCCCGACTACGTGC	CAGCGCTGAGTTGGTCCCCC	NM_013693.2
IL-1b	GCCAAGACAGGTCGCTCAGGG	CCCCCACACGTTGACAGCTAGG	NM_008361.3
F4/80//mEMR1	TGCATCTAGCAATGGACAGC	GCCTTCTGGATCCATTTGAA	NM_010130.4
iCAM	TTCTCATGCCGCACAGAACT	TCCTGGCCTCGGAGACATTA	NM_010493.3
α-sma/ACTA2	ACTACTGCCGAGCGTGAGAT	CCAATGAAAGATGGCTGGAA	NM_007392.2
C/EBP	TTGATGCAATCCGGATCAAACG	CAGTTACACGTGTGTTGCGTC	NM_001287738.1
18s rRNA	AACTTTCGATGGTAGTCGCCGT	TCCTTGGATGTGGTAGCCGTTT	NR_003278.3
GAPDH	ACAGTCCATGCCATCACTGC	GATCCACGACGGACACATTG	NM_008084.2
LPAR1	TCGCCTTCTTTTATAACCGG	TGATGCCCAGTCCCATC	NC_000070.7
LPAR2	GACCACACTCAGCTAGTCAAG	CTTACAGTCCAGGCCATCCA	NC_000074.7
LPAR3	TAACTCCCTGGTCATTGCTG	TTCCGGCGAAATCC	NC_000069.7
LPAR4	CAGTGCCTCCCTGTTTGTCTTC	GAGAGGGCCAGGTTGGTGAT	NC_000086.8
LPAR5	ACTCCACGCTGGCTGTATATG	GRAGCCAAAGGCCTGGTATTC	NC_000072.7
LPAR6	ACAGTGATGGGAGGAAGTGC	CCGCTGGAAAGTTCTCAAAG	NC_000080.7
**Human Primers**			
CCl2	GATCTCAGTGCAGAGGCTCG	TTTGCTTGTCCAGGTGGTCC	NM_002982.3
iNOS	CGCAGAGAACTCAGCCTCAT	TGCCTTGAGAACTTCGGGAC	NM_000625.4
IL-6	TGCAATAACCACCCCTGACC	ATTTGCCGAAGAGCCCTCAG	NM_000600.3
α-SMA/ACTA2	CCCCATCTATGAGGGCTATG	CAGTGGCCATCTCATTTTCA	NM_001613.2
col1a1	GTACTGGATTGACCCCAACC	CGCCATACTCGAACTGGAAT	NM_000088.3
PDGFβR	CATGGGGGTATGGTTTTGTC	GTAAGGTGCCAACCTGCAAT	NM_002609.4

GAPDH	TCCAAAATCAAGTGGGGCGA	TGATGACCCTTTTGGCTCCC	NM_001256799.1
RPS18	TGAGGTGGAACGTGTGATCA	CCTCTATGGGCCCGAATCTT	NM_022551.2
ENPP2	ATTACAGCCACCAAGCAAGG	TCCCTCAGAGGATTTGTCAT	NC_000008.11
LPAR1	AATCGGGATACCATGATGAGT	CCAGGAGTCCAGCAGATGATA	NC_000009.12
LPAR2	CGCTCAGCCTGGTCAAGACT	TTGCAGGACTCACAGCCTAAAC	NC_000019.10
LPAR3	AGGACACCCATGAAGCTAATGAA	GCCGTCGAGGAGCAGAAC	NC_000001.11
LPAR4	CCTAGTCCTCAGTGGCGGTATT	CCTTCAAAGCAGGTGGTGGTT	NC_000023.11
LPAR5	CCAGCACCTGCTCTTCAC	CCAGTGGTGCAGTGCGCGTAGT	NC_000012.12
LPAR6	AAACTGGTCTGTCAGGAGAAG	CAGGCAGCAGATTCATTGTCA	NC_000013.11
