# Supplementary material for: A type IV Autotaxin inhibitor ameliorates acute liver injury and nonalcoholic steatohepatitis
Source: EMBO Mol Med. 2022 Jul 14;14(9):e16333. doi: 10.15252/emmm.202216333 (PMC9449594; doi:10.15252/emmm.202216333)
Supplement: Supplementary file 2 — Expanded View Figures PDF [file EMMM-14-e16333-s009.pdf]

Expanded View Figures

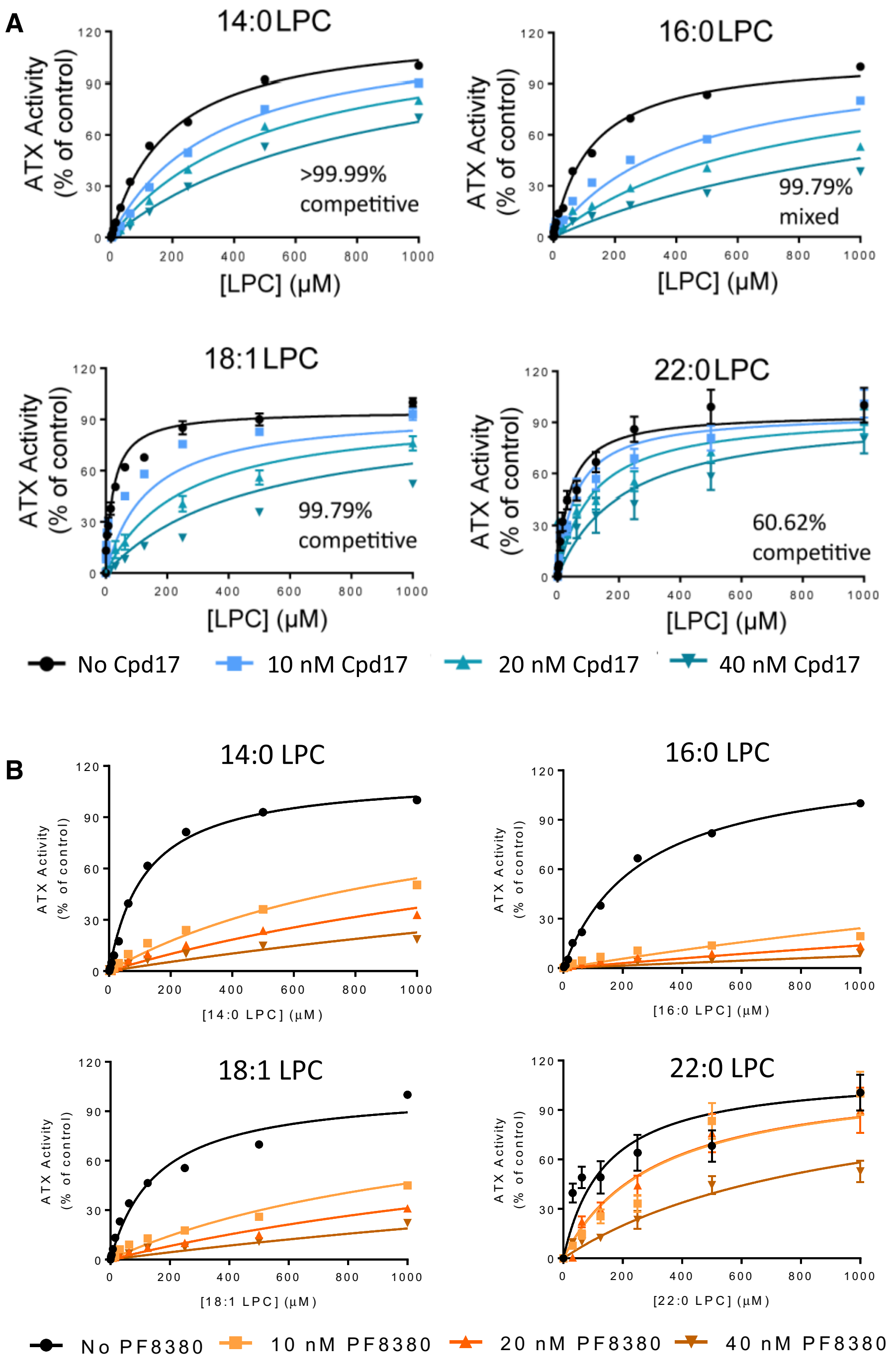

Figure EV1. Cpd17 and PF8380 inhibit ATX activity over different LPC species.

A, B Cpd17 (A) and PF8380 (B) were incubated with 20nM ATX for at least 30 min, upon which mixes were added to plates containing LPC and the reaction buffer for detection of choline release. Reaction kinetics were followed over time. Activity slopes were quantitated by linear regression and plotted versus LPC concentration. Bars represent the Mean ± SEM, $n = 3$. Quantitation of the type of inhibition was performed by comparison of non-linear regressions to competitive and non-competitive modes of inhibition.

© 2022 The Authors

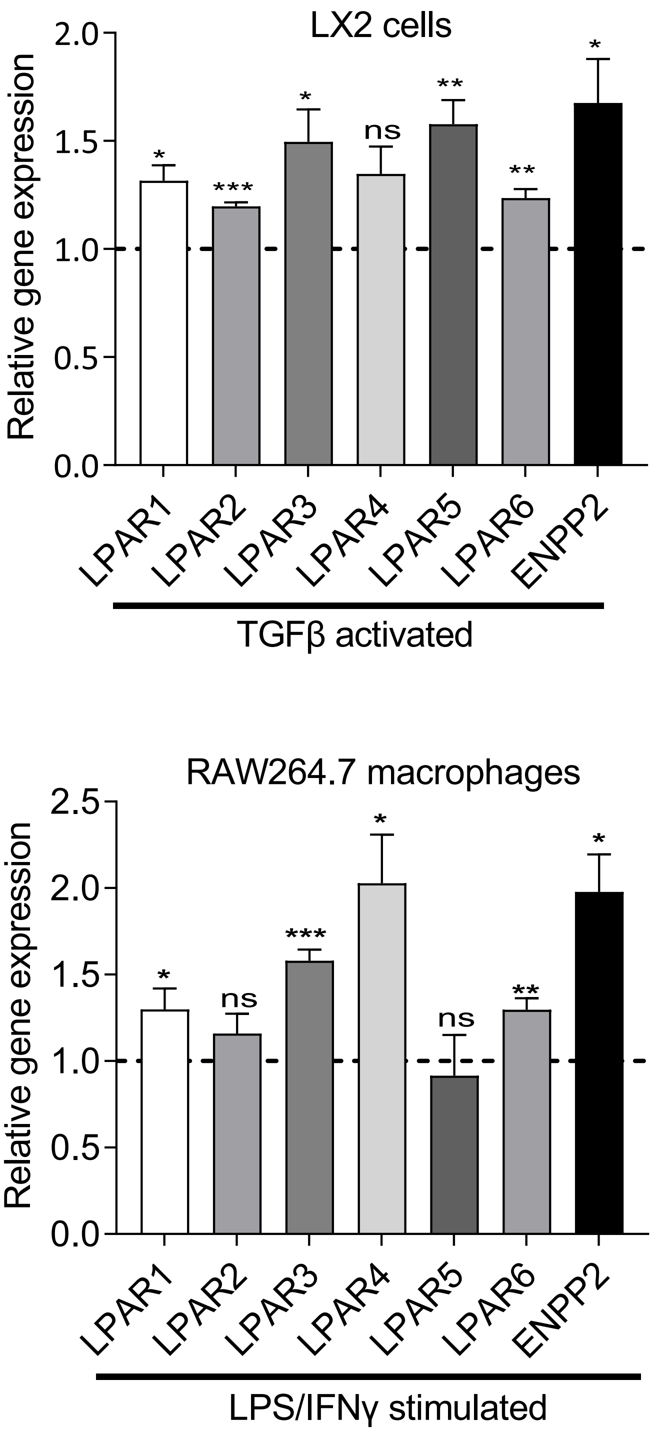

Figure EV2. The ATX/LPA pathway related genes (ENPP2 and LPAR$_{1\text{-}6}$) are upregulated in activated LX2 cells and pro-inflammatory RAW264.7 macrophages.

Relative LPAR$_{1\text{-}6}$ and ENPP2 gene expression (normalized with GAPDH) in TGFβ-activated LX2 cells versus control (top) and in LPS/IFNγ stimulated M1 RAW264.7 macrophages versus control (bottom). Bars represent the Mean ± SEM, $n = 3$. Statistical analysis was performed by two-tailed students *t*-test with Welch's correction. Results are normalized with respective control (non-activated/ unstimulated) cells depicted by dashed lines in both graphs. *$P < 0.05$, **$P < 0.01$, ***$P < 0.001$ denotes significance versus control (non-stimulated cells). ns = non-significant.

 © 2022 The Authors

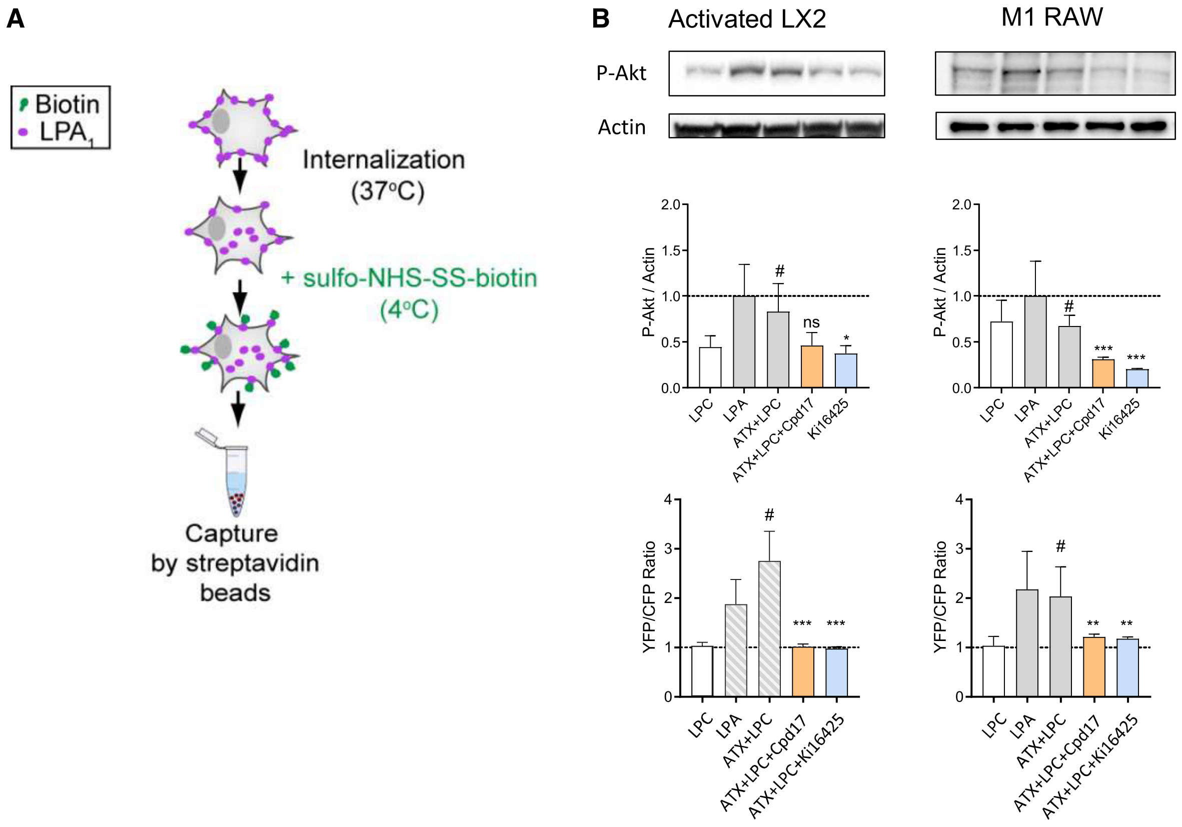

Figure EV3. ATX inhibition by Cpd17 resembles antagonism of LPA$_1$ by Ki16425.

A Schematic procedure of LPA$_1$ internalization assay for quantitation of surface localization. Data shown in Fig 5D.

B Representative Western-blot images and quantitation of pAKT normalized to β-actin. Serum-starved TGFβ-activated LX2 cells and M1 (LPS + IFNγ)-polarized RAW264.7 macrophages were treated for 10 min with the stimulants as indicated in the graphs. Bottom plots show the quantitation of RhoA activation from time-course stimulation of activated and serum-starved LX2 and M1-polarized RAW264.7 macrophages. RhoA activation is measured as a YFP/CFP fluorescent ratio. Bars represent the Mean + SEM, $n = 3$. Statistical analysis was performed by one-way analysis of variance (ANOVA) with Bonferroni *post-hoc* test; $^{\#}P < 0.05$ denotes significance versus control (non-stimulated cells); $^{*}P < 0.05$, $^{**}P < 0.01$, $^{***}P < 0.001$ denotes significance versus ATX + LPC treated cells. ns = non-significant.

Source data are available online for this figure.

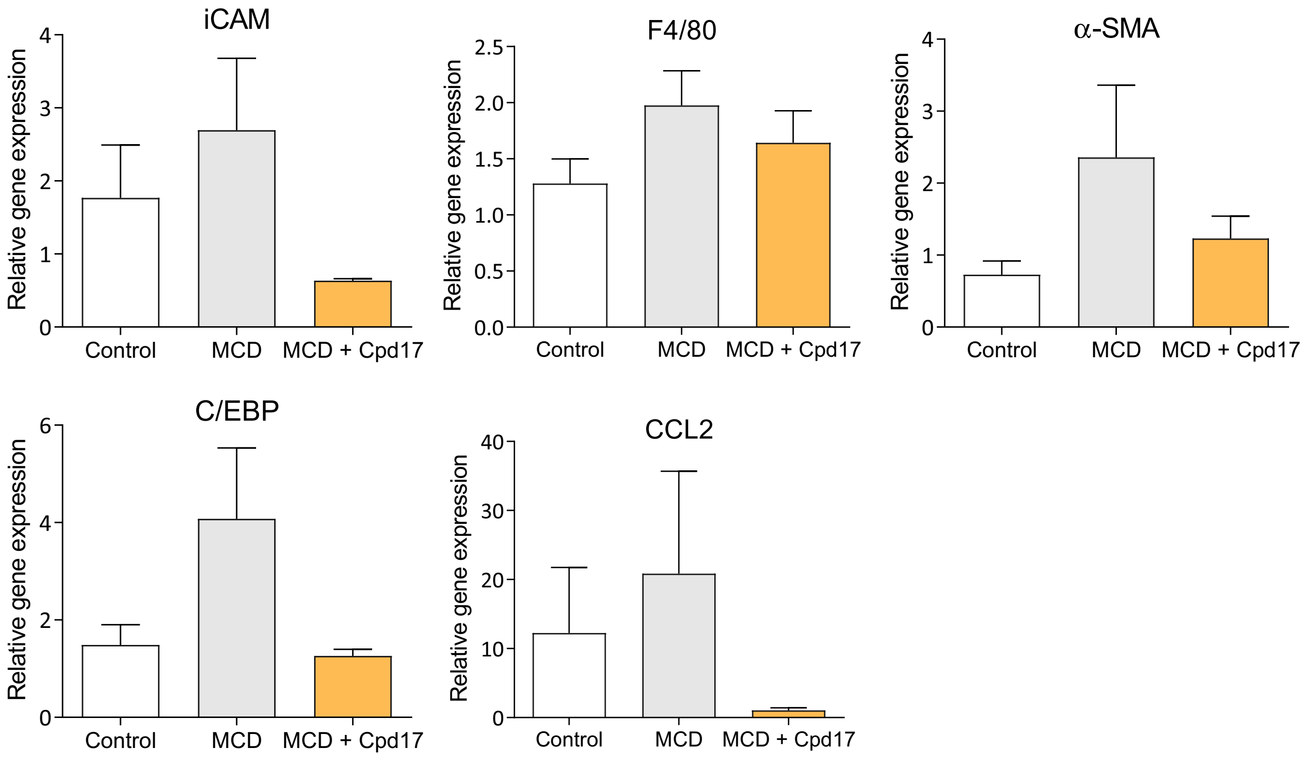

Figure EV4. Effect of Cpd17 on NASH related genes in MCD-mouse model.

Relative F4/80, CCL2, iCAM, α-SMA and C/EBP gene expression levels (normalized with GAPDH) as analyzed in control (n = 5), MCD (n = 6) and MCD + Cpd17 (n = 6) treated mouse livers. Bars represent the Mean + SEM.

© 2022 The Authors

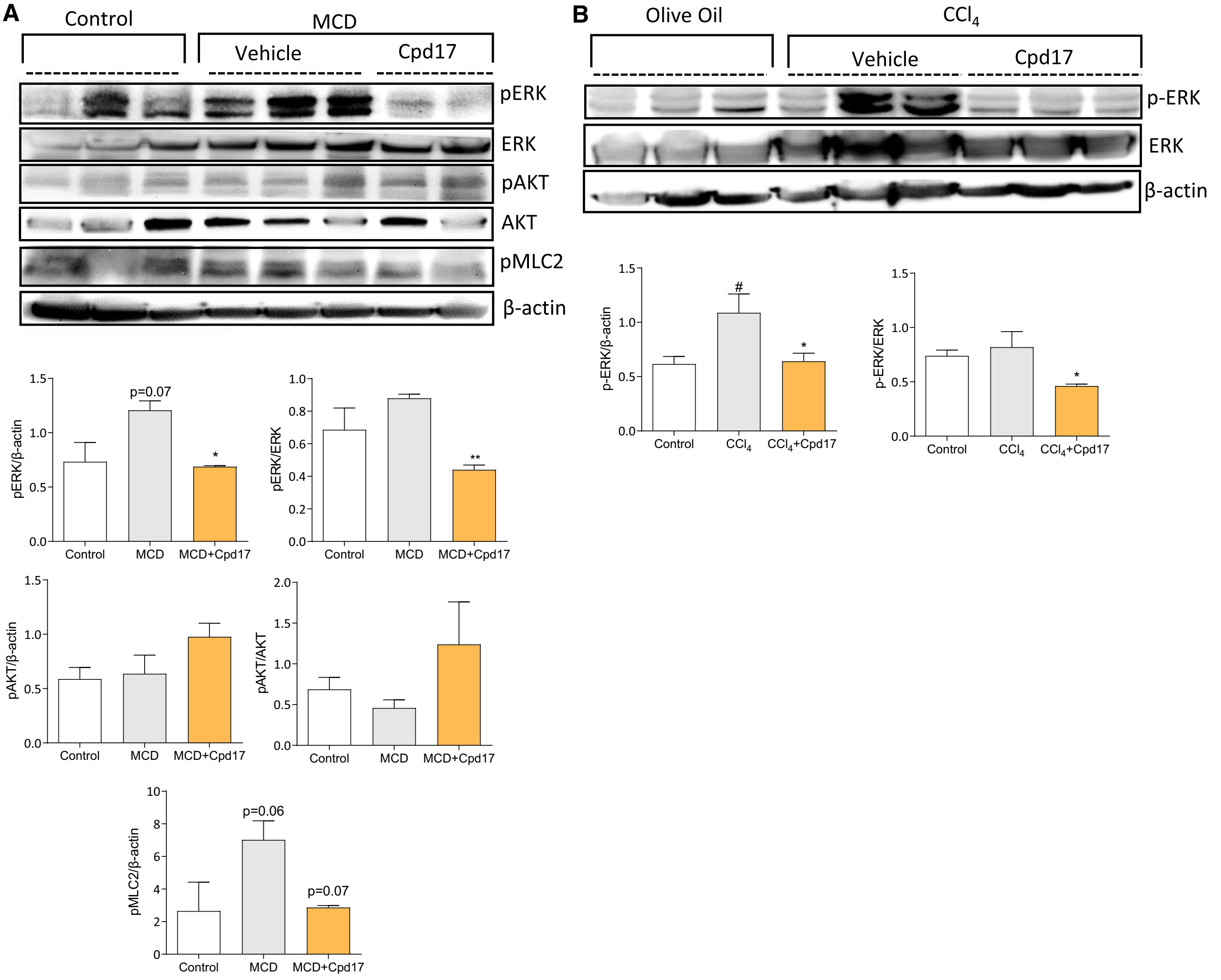

Figure EV5. Cpd17 differentially regulate downstream signalling *in vivo* in MCD-diet-induced NASH mouse model and in CCl_4-induced liver injury mouse model.

A Representative Western-blot images and quantitation of pERK normalized to ERK or β-actin as analyzed in control ($n = 5$), CCl_4 ($n = 5$) and CCl_4 + Cpd17 ($n = 4$) mouse livers. Bars represent the Mean + SEM; Statistical analysis was performed by one-way analysis of variance (ANOVA) with Bonferroni *post-hoc* test; $^{\#}P < 0.05$ denotes significance versus control (healthy mice); $^{*}P < 0.05$ denotes significance versus CCl_4 mice.

B Representative Western-blot images and quantitation of pERK, pAKT, pMLC2 normalized to ERK, AKT or β-actin as analyzed in control ($n = 5$), MCD ($n = 6$) and MCD + Cpd17 ($n = 6$) mouse livers. Bars represent the Mean + SEM. Statistical analysis was performed by one-way analysis of variance (ANOVA) with Bonferroni *post-hoc* test. $P = 0.07$, $P = 0.06$ denotes significance versus control (healthy mice); $^{*}P < 0.05$, $^{**}P < 0.01$, $P = 0.07$ denotes significance versus MCD-diet fed mice.

Source data are available online for this figure.
