# Supplementary figures and images for: A type IV Autotaxin inhibitor ameliorates acute liver injury and nonalcoholic steatohepatitis
Source: EMBO Mol Med. 2022 Jul 14;14(9):e16333. doi: 10.15252/emmm.202216333 (PMC9449594; doi:10.15252/emmm.202216333)

Figure EV5

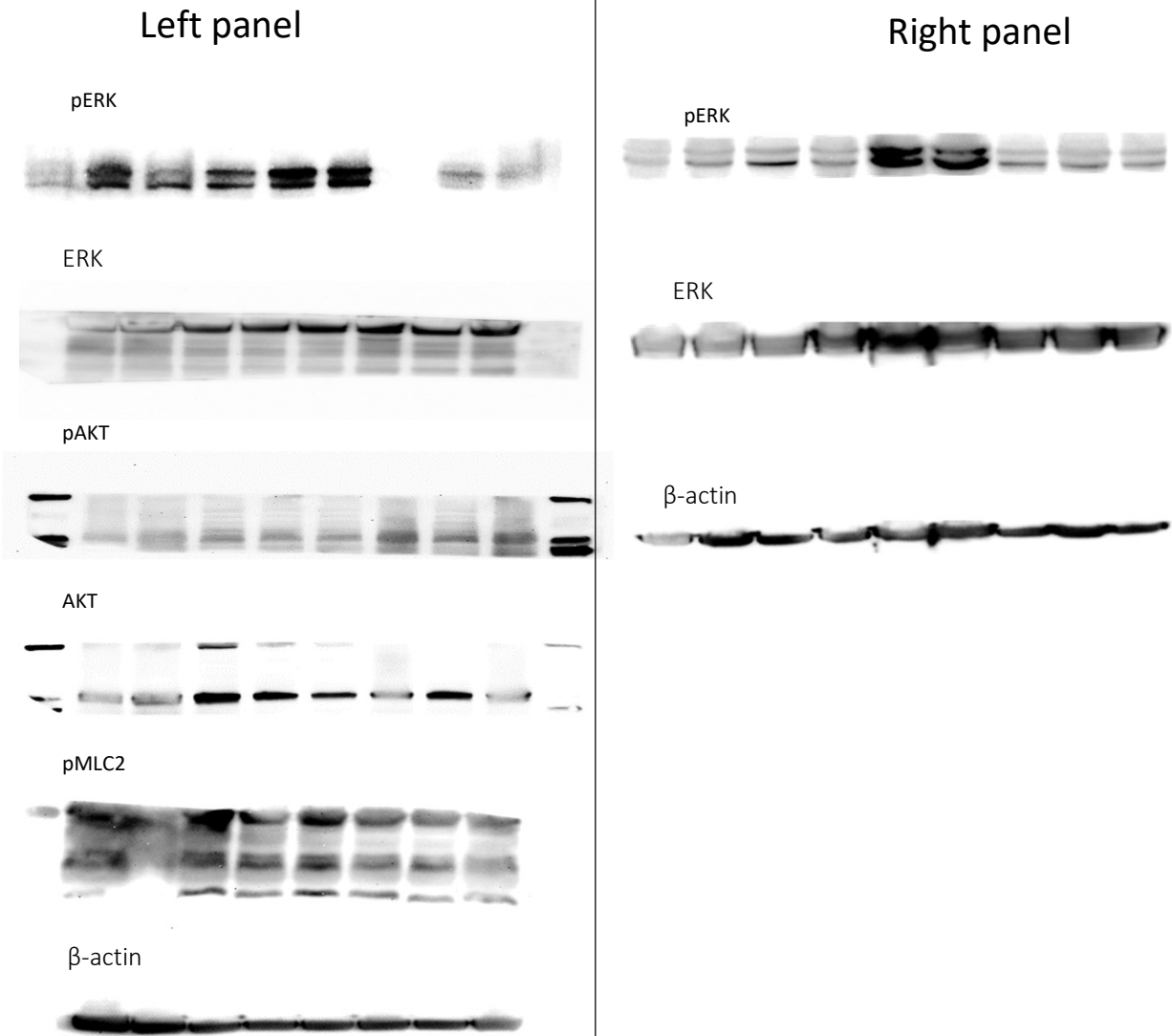

Supplement: Supplementary file 3 — Source Data for Expanded View [file EMMM-14-e16333-s006.zip › Uncropped_images_FigureEV5.pdf]

Figure EV3B

Left panel

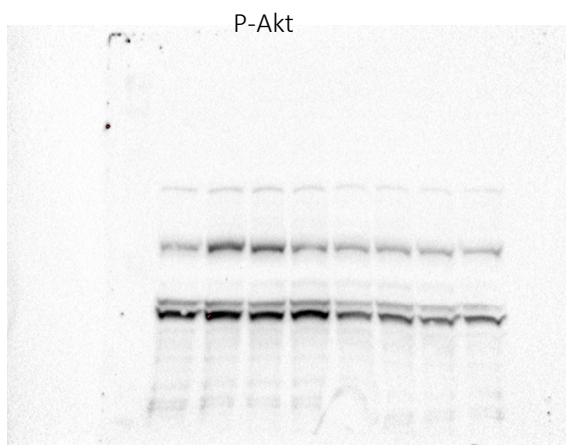

Right panel

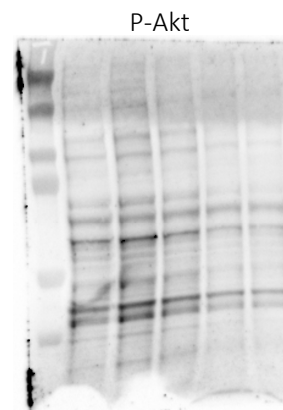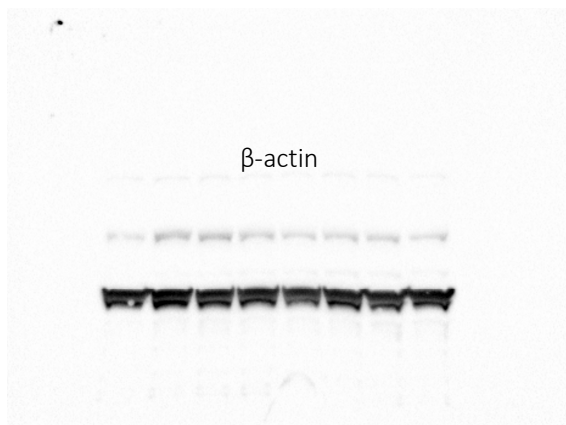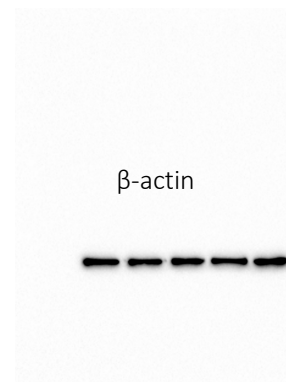

Supplement: Supplementary file 3 — Source Data for Expanded View [file EMMM-14-e16333-s006.zip › Uncropped_images_FigureEV3.pdf]

Figure 1

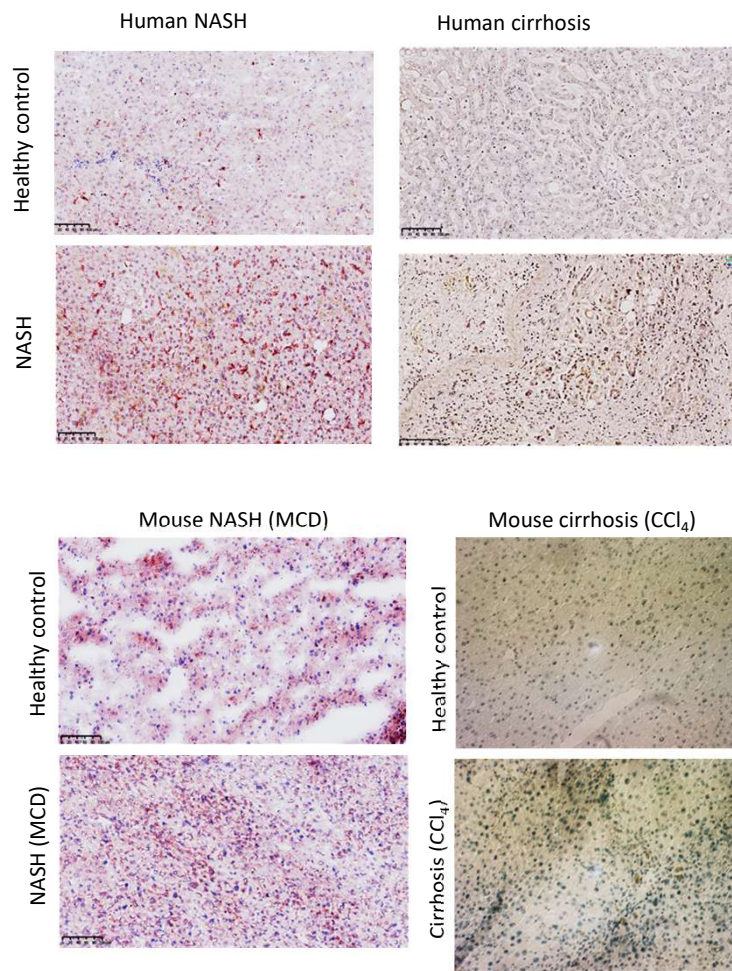

Supplement: Supplementary file 4 — Source Data for Figure 1 [file EMMM-14-e16333-s003.pdf]

Figure 3A

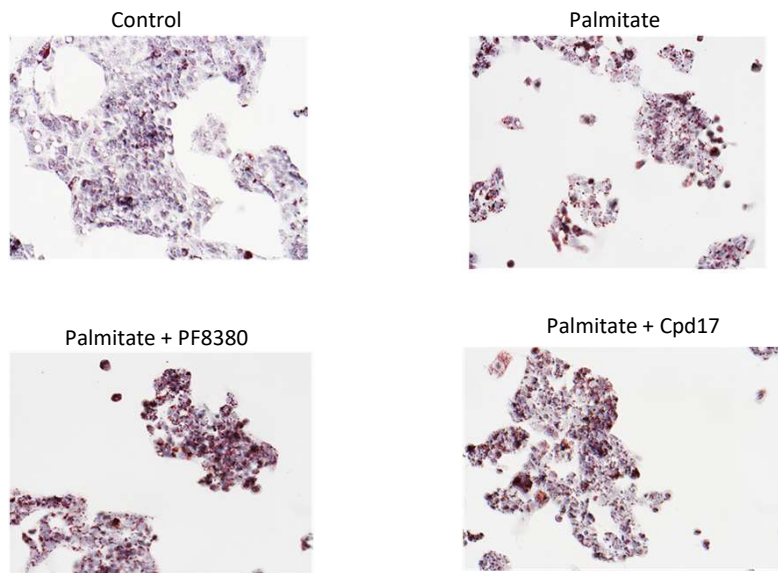

Supplement: Supplementary file 5 — Source Data for Figure 3 [file EMMM-14-e16333-s005.pdf]

Figure 4

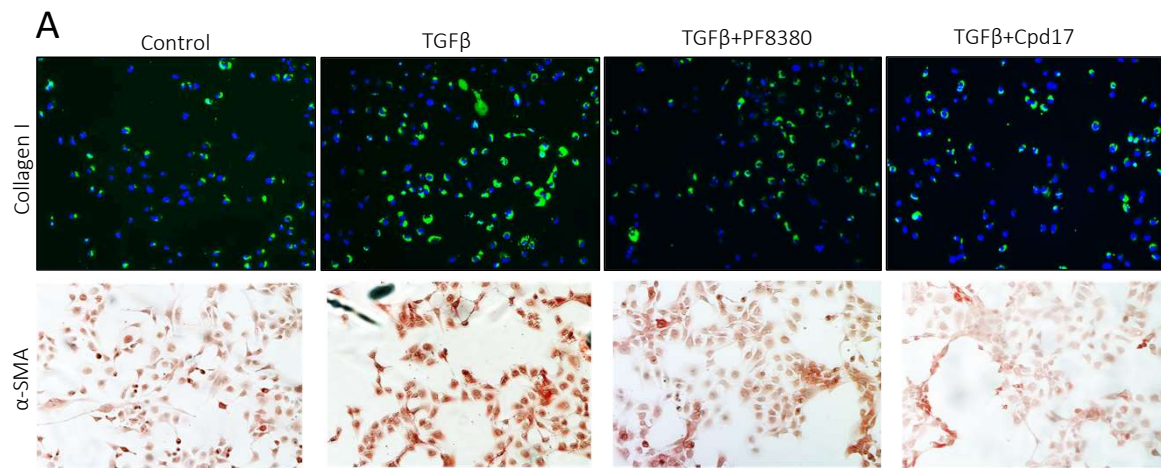

Supplement: Supplementary file 6 — Source Data for Figure 4 [file EMMM-14-e16333-s004.pdf]

Figure 5c

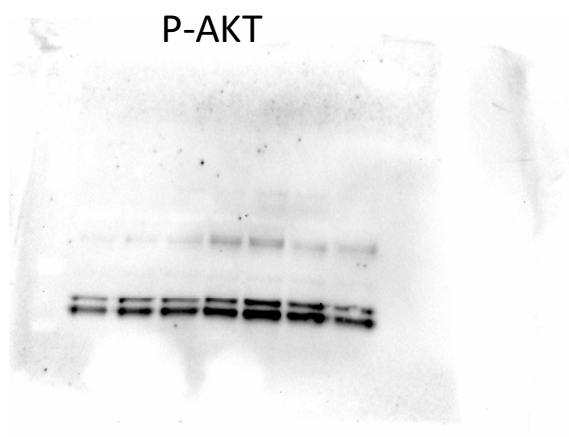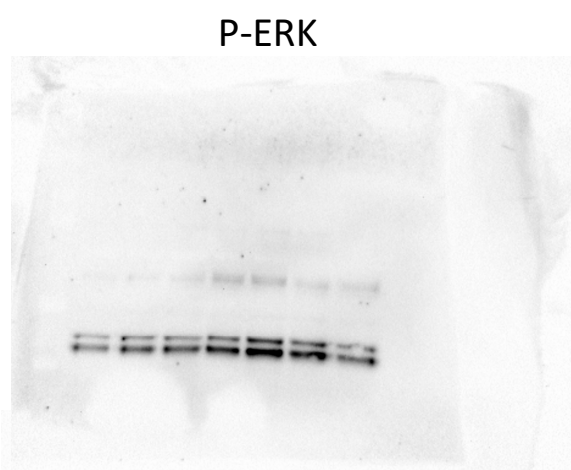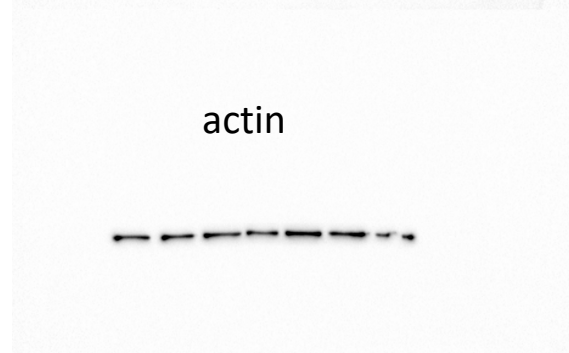

Supplement: Supplementary file 7 — Source Data for Figure 5 [file EMMM-14-e16333-s002.pdf]

Figure 6D

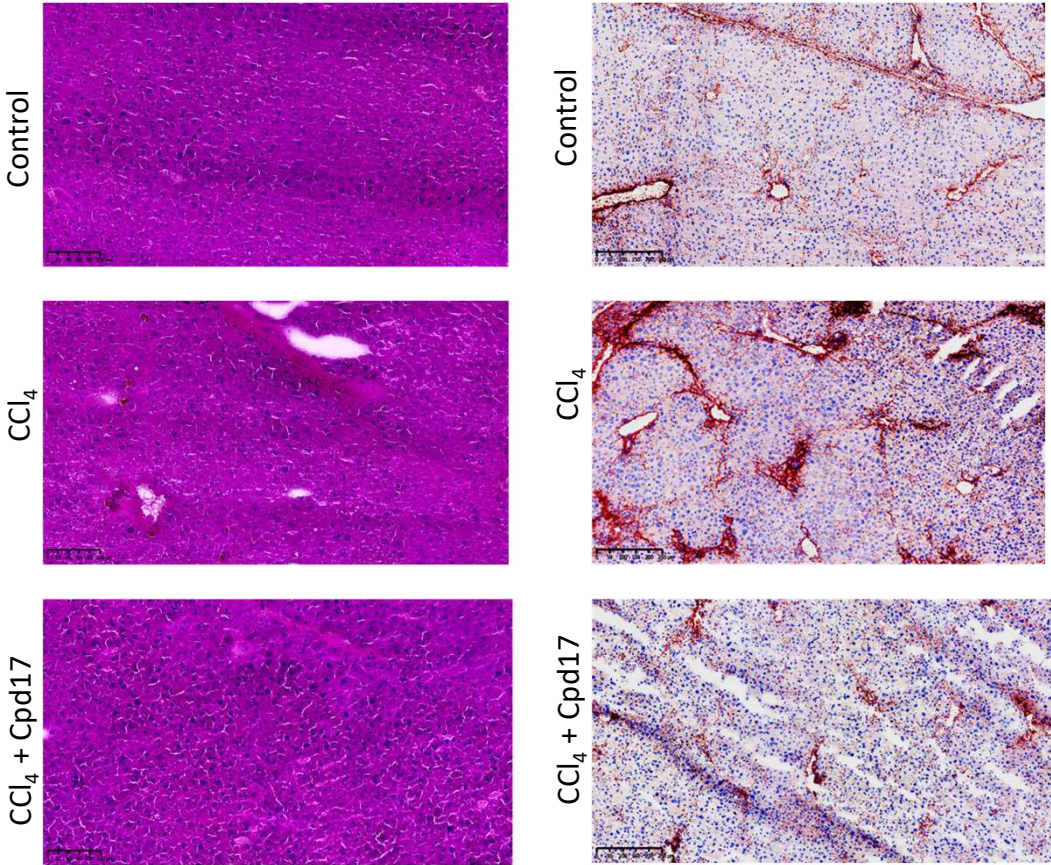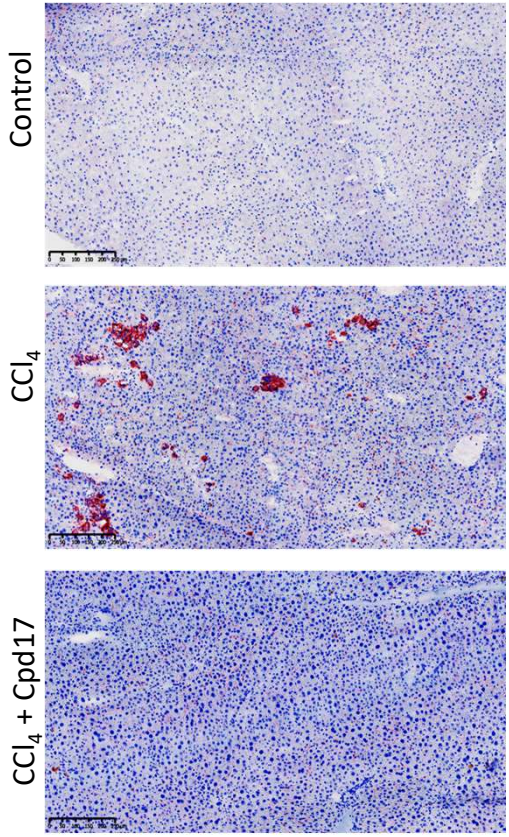

Figure 6D

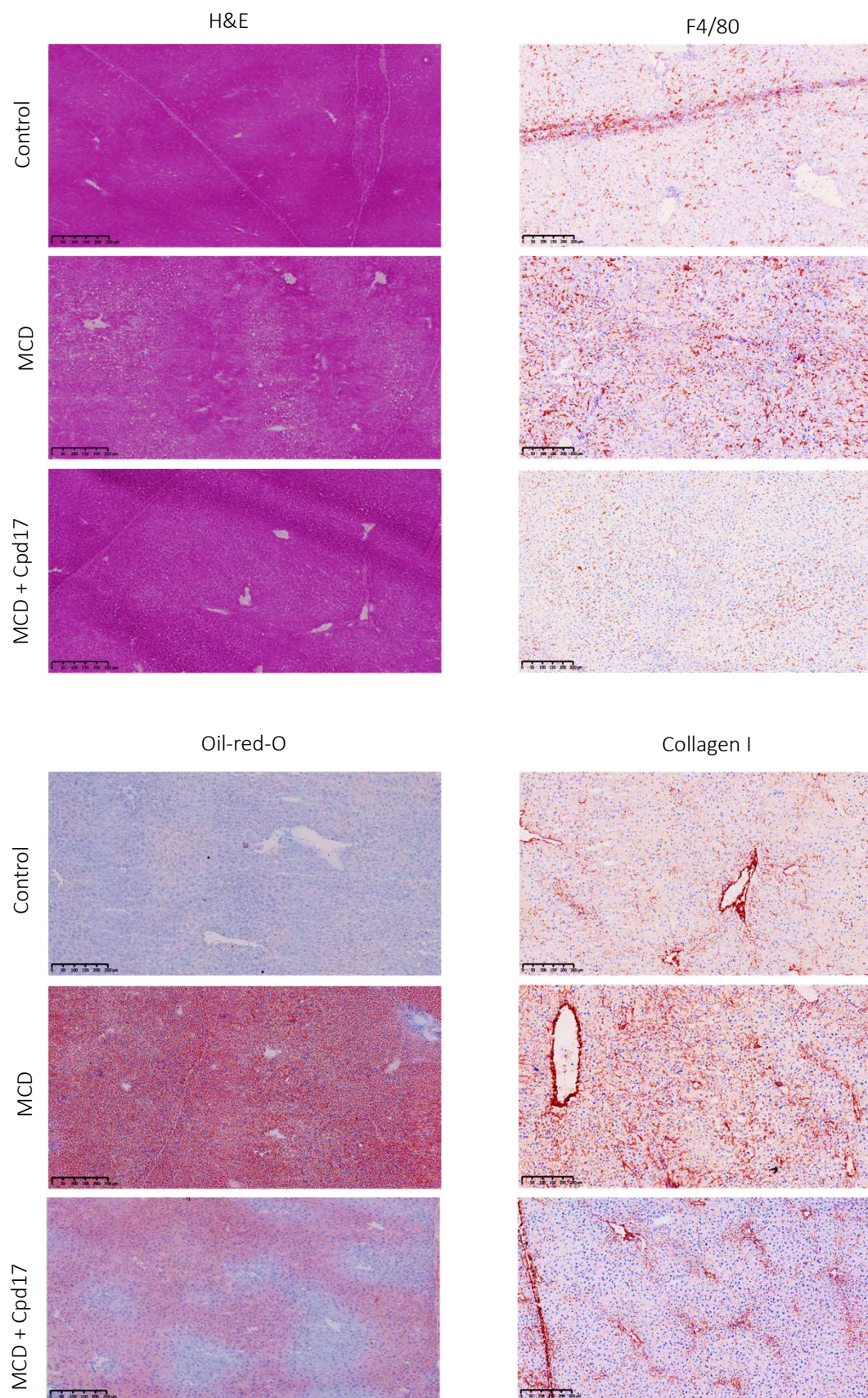

Supplement: Supplementary file 8 — Source Data for Figure 6 [file EMMM-14-e16333-s008.pdf]
