# Supplementary material for: A type IV Autotaxin inhibitor ameliorates acute liver injury and nonalcoholic steatohepatitis
Source: EMBO Mol Med. 2022 Jul 14;14(9):e16333. doi: 10.15252/emmm.202216333 (PMC9449594; doi:10.15252/emmm.202216333)
Supplement: Supplementary file 9 [file EMMM-14-e16333-s007.pdf]

EMBO Molecular Medicine

A type IV Autotaxin inhibitor ameliorates acute liver injury and non-alcoholic steatohepatitis

Richell Booijink, Fernando Salgado-Polo, Craig Jamieson, Anastassis Perrakis, and Ruchi Bansal
DOI: 10.15252/emmm.202216333

Corresponding authors: Ruchi Bansal (r.bansal@utwente.nl) , Anastassis Perrakis (a.perrakis@nki.nl)

Review Timeline:

Transferred from Review Commons:	18th May 22
Editorial Decision:	9th Jun 22
Revision Received:	29th Jun 22
Accepted:	30th Jun 22

Editor: Zeljko Durdevic

Transaction Report:

This manuscript was transferred to EMBO Molecular Medicine following peer review at Review Commons.

(Note: With the exception of the correction of typographical or spelling errors that could be a source of ambiguity, letters and reports are not edited. Depending on transfer agreements, referee reports obtained elsewhere may or may not be included in this compilation. Referee reports are anonymous unless the Referee chooses to sign their reports.)

Review #1

1. Evidence, reproducibility and clarity:

Evidence, reproducibility and clarity (Required)

The study analyzed the role of autotaxin inhibitor in acute liver injury and NASH in mice.
It delineates the evidence in the signaling pathway from LPA to Autotaxin and confirms the findings using a different inhibitor classes as well. It's finally provides in vivo evidence for the use of type IV inhibitor.
Studies well performed, the manuscript is well written and well presented. It includes in vitro and in vivo evidence and therefore it is a state of the art pharmacological study.

Major comments:

1. The previous evidence has already worked on different models, and therefore the present it data are complementary to the existing evidence but it's also means that originality of the concept is limited, Still I believe that the authors can work out a little better potential original findings in this manuscript which has not been published before.
2. It is puzzling me that the previous described models of combining CCL4 with western diet we're not used, but the very aggressive MCD model without obesity was used here. Can the authors comment why they use this model?
3. Can the authors deliver evidence with CCL 4 plus WD model? Is there any issue with increase body weight and autotaxin, or adipose tissue distribution?
4. Also it is astonishing that show little time injection of CCL4 leads to on my opinion high collagen accumulation in the staining? Is this only after four times of injury?
5. The study is very complex since the authors are analyzing different cell types of the liver and it's remains unclear which of them has really the main role in injury or improvement of damage

Minor comments:

1. It is difficult to understand how Autotaxin inhibitor impairs RhoA/rock activation, Maybe some citations analyze this in hepatic stellate cells
2. The original publications for animal models should be cited and not the reviews

2. Significance:

Significance (Required)

This manuscript has clinical implications since different targets for treatment of NASH have failed in clinical studies. Nevertheless the originality of the study it's rather limited by this complementary and important for the fields due to the clinical significance.

3. How much time do you estimate the authors will need to complete the suggested revisions:

Estimated time to Complete Revisions (Required)

(Decision Recommendation)

Between 1 and 3 months

Review #2

1. Evidence, reproducibility and clarity:

Evidence, reproducibility and clarity (Required)

I think Cpd17 will be a candidate interesting drug to be considered with NASH and acute liver injury.

To clear the effect of Cpd17, dose dependent experiment is needed.

Fig 3e is not clear. Please revise this.

2. Significance:

Significance (Required)

Cpd17 will be a new drug. To confirm this, author should revise the paper to analyze the in vivo mechanism how Cpd17 regulate acute liver injury and NASH. In vitro analysis is clear.

3. How much time do you estimate the authors will need to complete the suggested revisions:

Estimated time to Complete Revisions (Required)

(Decision Recommendation)

Between 3 and 6 months

Review #3

1. Evidence, reproducibility and clarity:

Evidence, reproducibility and clarity (Required)

In this study, the authors aimed to evaluate the therapeutic potential of a type IV ATX inhibitor, Cpd17, in liver injury. They first confirmed the involvement of the ATX/LPA signaling axis in human and murine diseased livers. Then, they evaluated the effects of Cpd17, in comparison with a classic type I ATX inhibitor PF8380, in vitro. While both inhibitors attenuated induced liver cells injury phenotypes, Cpd17 exhibit higher potency in inhibiting downstream LPA signaling pathways. Finally, the authors demonstrated that Cpd17 has an excellent potential for reducing liver injury in both CCl4-induced acute liver injury and diet-induced NASH mouse models in vivo. Although data provided here by the authors could have interesting implications, there are several concerns and problems in research design. Specific points to be considered are listed below:

1. The authors only claimed peroxidase activity was developed using AEC substrate (red color) kit for immunohistochemistry in Materials and methods. Therefore, the authors should also provide brown color substrate information for some immunohistochemistry images (figure 1 C-D).
2. The authors claimed "ATX inhibition by both Cpd17 and PF8380 reduced lipid accumulation in hepatocytes as assessed using oil-red-o staining (Fig. 3a)." However, these changes were not significant.
3. The authors mentioned that "Cpd17, but not PF8380, strongly inhibited the ATX+LPC(LPA)-induced RhoA activation in LX2 cells (Fig. 5b)." However, it is not clear what the difference is between groups of ATX+LPC and LPA in figure 5.
4. The same staining was not quantified in Figure 6b but quantified in Figure 7c, 7e and 7f. The authors should be consistent with the quantification.
5. The authors should improve the staining picture quality and background, especially the strong red positive reaction in the H&E staining in Figure 6b is very strange.
6. It is too simplistic to assess only the phenotype in in vivo studies, and authors should also evaluate the RhoA signaling pathway in vivo to confirm conclusion from in vitro studies.

2. Significance:

Significance (Required)

A type IV Autotaxin inhibitor has an excellent potential for clinical application in liver diseases.

3. How much time do you estimate the authors will need to complete the suggested revisions:

Estimated time to Complete Revisions (Required)

(Decision Recommendation)

Between 3 and 6 months

Full Revision

Manuscript number: RC-2021-01201 *(Revision following submission to Review Commons)*

Manuscript title: A type IV Autotaxin inhibitor ameliorates acute liver injury and non-alcoholic steatohepatitis in mice

Authors: Richell Booijink, Fernando Salgado-Polo, Craig Jamieson, Anastassis Perrakis†, Ruchi Bansal†

†These authors contributed equally to this work

Corresponding authors: Anastassis Perrakis (a.perrakis@nki.nl); Ruchi Bansal (r.bansal@utwente.nl)

General comments/next steps

1. REVISION & RESPONSE TO REVIEWERS: There are 2 choices (see details: https://www.reviewcommons.org/authors/#submitting-to-affiliate):

- upload within *four weeks* a *preliminary revision* accompanied by a *revision plan* (download template: https://reviewcommons.org/templates/revision-plan.docx). Please use this template when submitting your preliminary revision.

- or *request more time* via email to perform a *full revision* (download template: https://reviewcommons.org/templates/full-revision.docx). Please use this template when submitting your full revision.

*Response: We opted for the *full revision* and requested more time. We have used the provided template for submitting the full revision.*

2. POSTING THE PREPRINT: when submitting your revision, the system will require you to post it as a preprint if this has not yet been done. Posting the preprint is mandatory before transferring your manuscript to an affiliated journal. *Please note only the preprint will be posted. Posting the reviews remains optional.

Response: We have posted the manuscript as a preprint while submitting the revision.

3. REVIEWS ON BIORXIV or MEDRXIV: We strongly encourage the posting of your reviews on BioRxiv and MedRxiv. You will be able to instruct Review Commons to post the reviews and your response alongside your preprint. Please follow the instructions that are provided online on the submission screen.

Response: We chose not to post the reviews and our response alongside the preprint

4. TRANSFER TO AFFILIATE JOURNALS: When you submit the preliminary/full revision, you will be presented with a screen that allows you to select the affiliate journal to which you want to submit. Please note that the full set of reviews will be transferred alongside your response (revision plan or full revision) and the (preliminarily or fully) revised manuscript.

Response: We have submitted the revised manuscript along with our responses to EMBO Molecular Medicine.

5. EVALUATION AT AFFILIATES: Upon transfer, the recipient journal will consider the manuscript as a formal submission and use the transferred reviews and your response/revision plan to make an initial decision. Note that while it is under consideration at an affiliate journal, the manuscript cannot be transferred to any other affiliates.

Response: Thank you for this information. We will wait for the decision after transferring our manuscript.

6. NEW ROUND OF TRANSFER: If the first journal declines, you will be directly informed by this journal. The manuscript will then be unlocked on the Review Commons site so that you can initiate up to two additional rounds of transfer.

Response: Thank you for this information.

Reviewer: 1

General Comments

The study analyzed the role of autotaxin inhibitor in acute liver injury and NASH in mice. It delineates the evidence in the signaling pathway from LPA to Autotaxin and confirms the findings using a different inhibitor classes as well. It's finally provides in vivo evidence for the use of type IV inhibitor. Studies well performed, the manuscript is well written and well presented. It includes in vitro and in vivo evidence and therefore it is a state-of-the-art pharmacological study.

Significance: This manuscript has clinical implications since different targets for treatment of NASH have failed in clinical studies. Nevertheless, the originality of the study it's rather limited by this complementary and important for the fields due to the clinical significance.

Response: We thank the reviewer for acknowledging the quality of the work and the manuscript, and for providing constructive comments. A highly original component of our work is the use of the two inhibitor classes in relevant cell systems, to confirm that Type IV inhibition has more profound effects. The mechanism underlying the in vivo superiority of Type IV inhibitors (in the lung) is now available as a pre-print (https://www.biorxiv.org/content/10.1101/2022.04.09.487723v1). This work is building on this, re-enforcing, and extending on these new mechanistic insights, while presenting new encouraging results relevant for liver disease.

Point-by-point description of the revisions:

Major comments:

1. The previous evidence has already worked on different models, and therefore the presented data are complementary to the existing evidence, but it's also means that originality of the concept is limited, Still I believe that the authors can work out a little better potential original finding in this manuscript which has not been published before.

The novelty and motivation behind our work is in the use of inhibitors with a different mechanism, but also in the comprehensive study of two disease models. These are also discussed in the introduction section (refer to page no. 5 and line nos. 6-19).

Previous studies have used different ATX inhibitors, mainly type I (PF8380) and type III (PAT-505) and have indeed shown beneficial effects of ATX inhibition in liver fibrosis and cancer. However, type IV ATX inhibitors have not been explored in liver diseases. In this study, the type IV inhibitor (Cpd17) that occupies the orthosteric lipid-binding pocket and the allosteric tunnel of ATX, but does not interact with the catalytic site, is used. This is the first time a comparison between a type I and IV inhibitors is done in cell-based assays relevant for liver disease. We note that the ***inhibitor type choice is extremely important for inhibiting ATX function****. All ATX inhibitors entering clinical trials (in patients with pulmonary fibrosis or cancer) occupy the allosteric tunnel. These include ziritaxestat (Galapagos B.V. Belgium), which progressed to phase 3; BBT-877 (Bridge Biotherapeutics Inc., Pangyo, South Korea), IOA-289 (iOnctura SA, Geneva, Switzerland), and cudetaxestat (Blade Therapeutics Inc., South San Francisco, USA). None of the potent type I orthosteric ATX inhibitors have entered clinical trials, to the best of our knowledge. This is the first time that an inhibitor type IV with real clinical promise for ATX inhibition has been evaluated in liver disease models, CCl_4-induced liver injury and MCD-diet induced NASH mouse models.*

2. It is puzzling me that the previous described models of combining CCL4 with western diet were not used, but the very aggressive MCD model without obesity was used here. Can the authors comment why they use this model?

The MCD model has the advantage of being more efficient and reproducible for inducing severe liver damage and progressive fibrosis. Moreover, MCD diet-fed rodents display mechanisms that have been implicated in human NASH progression. Thus, this diet approach models the subgroup of NASH patients with histologically advanced NASH and is ideal for studying mechanisms driving NASH-related inflammation/fibrosis, as well as strategies to inhibit these processes. In contrast, the Western diet model with and without hepatotoxins (CCl_4) mimics obese NAFLD/NASH patients i.e., insulin resistance and metabolic syndrome but relatively mild liver injury. The Western diet model is useful for studying NAFLD/NASH associated with systemic metabolic and cardiovascular risk related to type 2 diabetes and atherosclerosis. As liver injury and fibrosis are relatively mild and slowly progressive in the western diet models, we opted for the MCD-diet model for studying the therapeutic efficacy of our inhibitor.

3. Can the authors deliver evidence with CCL 4 plus WD model? Is there any issue with increase body weight and autotaxin, or adipose tissue distribution?

We have already performed the study in the MCD-diet model, and it is regrettably practically not possible to convince animal ethics committee to perform another study in another model of NASH. Therefore, it is not possible to provide evidence in a CCl_4 plus WD model. We also note that previous studies have reported beneficial effects of ATX inhibition in different types of NASH models, including CDAA NASH model and/or STAM model; there are no studies with the high fat/WD (with or without CCl_4) NASH model. One of the reasons behind this (as suggested by the reviewer) could be that high-fat diets exacerbate pathophysiological events associated with dysregulation of LPA metabolism and signalling. Moreover, it has been shown that the ATX-LPA pathway impacts obesity and obesity-associated disorders, including impaired glucose homeostasis, insulin resistance, and cardiovascular disease, which would complicate issues.

4. Also it is astonishing that show little time injection of CCL4 leads to on my opinion high collagen accumulation in the staining? Is this only after four times of injury?

It is indeed astonishing that the sensitivity of the rodents for CCl_4 has increased dramatically over the years; we have seen that in our own experience, and it is also supported by anecdotal evidence by others. We have seen that the previously tolerated dose of 1ml/kg CCl_4 (now a lethal dose) is equivalent to a single injection of 0.2 ml/kg CCl_4. In this study, we administered a single injection of 0.5 ml/kg CCl_4 which resulted in significantly high amount of fibrosis as can be seen in collagen staining, and in the liver to body weight ratio in this model.

5. The study is very complex since the authors are analyzing different cell types of the liver and it's remains unclear which of them has really the main role in injury or improvement of damage.

Liver disease is complex and different cell types play different roles in the disease process. In the introduction section (page 3, line 7-16), we tried to clarify the roles of different cell types in the disease progression. In this paper, we studied three major cell types i.e., (a) ***hepatocytes*** *which upon injury (by hepatotoxins or lipid-induced toxicity) initiate inflammatory response by increasing*

infiltration and activation of pro-inflammatory (b) ***macrophages*** *which activate (c)* ***hepatic stellate*** *cells that secrete extracellular matrix factors and result in fibrosis development. We show that Cpd17 reduced lipid accumulation in hepatocytes both in vitro and in vivo. More strikingly, Cpd17 inhibited activation of macrophages and hepatic stellate cells suggesting a direct effect on liver inflammation and fibrosis development as shown in in vitro and in vivo models.*

Minor comments:

1. It is difficult to understand how Autotaxin inhibitor impairs RhoA/rock activation, maybe some citations analyze this in hepatic stellate cells

This has been now included in the revised version of the manuscript.

2. The original publications for animal models should be cited and not the reviews

The original publications for animal models are now included in the revised version of the manuscript.

Reviewer: 2

General Comments

I think Cpd17 will be a candidate interesting drug to be considered with NASH and acute liver injury.

We are glad that the reviewer shares our enthusiasm for Cpd17.

Point-by-point description of the revisions:

Significance: Cpd17 will be a new drug. To confirm this, author should revise the paper to analyze the in vivo mechanism how Cpd17 regulate acute liver injury and NASH. In vitro analysis is clear.

The in vivo mechanism of action of Cpd17 is indeed interesting. As the in vitro analysis is rather clear, as the referee accepts, we decided to check the same pathways (pAKT, pERK and RhoA) as in vitro, also in vivo. However, it is important to note that these pathways are involved in several cellular processes independent of ATX signalling, and analysis of in vivo results in signalling is challenging. ***It is difficult to conclude if our observations are a direct effect of ATX inhibition or indirect effect.*** *Nevertheless, upon analysis, we observed increased ERK activation in both animal models and a significant decrease after treatment with Cpd17 (Supplementary Fig. 5 and 6), as also in vitro. However, we did not observe a significant effect on AKT activation: in contrast to our in vitro results, we observed (non-significant) increased AKT activation after Cpd17 treatment (Supplementary Fig. 5): this could be because we observe that in a mixed cells population. AKT* ***activation*** *is involved in restricting pro-inflammatory and promoting anti-inflammatory responses via negative regulation of TLR and NF-κB signalling in* ***macrophages****, while PI3K/AKT pathway* ***blockade*** *in HSCs has been shown to suppress fibrotic responses by inhibiting* ***hepatic stellate cells (HSCs)*** *proliferation and collagen synthesis. Finally, another study has suggested the ERK-AKT axis to act as a switch regulating liver regeneration or fibrosis via liver sinusoidal endothelial cells (LSECs)-HSCs communication, complicating direct analysis even more. In addition, RhoA signalling (analysed using pMLC2) increased in MCD-fed mice and decreased upon Cpd17 treatment; however, these observations were not statistically significant (Supplementary Fig. 5). No effect in AKT and RhoA pathways were observed in acute CCl_4-induced liver injury mouse model. Collectively though, these results demonstrate that ATX inhibition by Cpd17 in vivo inhibited key pathways that are involved in liver diseases, and strongly suggests that this inhibitor or other type IV inhibitors should be considered for the clinical application.*

These experiments complement the in vitro mechanistic analysis and are included in the revised manuscript. Please refer to supplementary figures 5 and 6; page 19, line 1-12, and page 21, line 6-18.

2. To clear the effect of Cpd17, dose dependent experiment is needed.

We have performed dose-dependent experiments in pilot studies, based on which we continued with 1μM dose of the inhibitors in in vitro studies and 5mg/kg in in vivo studies. As only limited experiments were performed to assess the dose for follow-up studies, we have not included these in the manuscript.

3. Fig 3e is not clear. Please revise this.

We think the reviewer is referring to figure 3a which we have revised in the new submitted version.

Reviewer: 3

General Comments

In this study, the authors aimed to evaluate the therapeutic potential of a type IV ATX inhibitor, Cpd17, in liver injury. They first confirmed the involvement of the ATX/LPA signaling axis in human and murine diseased livers. Then, they evaluated the effects of Cpd17, in comparison with a classic type I ATX inhibitor PF8380, in vitro. While both inhibitors attenuated induced liver cells injury phenotypes, Cpd17 exhibit higher potency in inhibiting downstream LPA signaling pathways. Finally, the authors demonstrated that Cpd17 has an excellent potential for reducing liver injury in both CCl_4-induced acute liver injury and diet-induced NASH mouse models in vivo.

Significance: A type IV Autotaxin inhibitor has an excellent potential for clinical application in liver diseases.

We are glad that the reviewer finds our study clinically promising.

Point-by-point description of the revisions:

Major comments:

Although data provided here by the authors could have interesting implications, there are several concerns and problems in research design. Specific points to be considered are listed below:

1. The authors only claimed peroxidase activity was developed using AEC substrate (red color) kit for immunohistochemistry in Materials and methods. Therefore, the authors should also provide brown color substrate information for some immunohistochemistry images (figure 1 C-D).

We thank the reviewer for noticing this. Indeed, both staining's have been performed using different substrates i.e., AEC or DAB. We have corrected this mistake in the revised version of the manuscript. Please refer to Materials and Methods section, page 10-11, and figure 1 figure legend.

2. The authors claimed "ATX inhibition by both Cpd17 and PF8380 reduced lipid accumulation in hepatocytes as assessed using oil-red-o staining (Fig. 3a)." However, these changes were not significant.

To address this point, we performed additional staining's and analysed the previous staining again. The differences are now significant for Cpd17-treatment as compared to PF8380-treated hepatocytes. These new results are now included in the revised version of the manuscript. Please refer to page 14, line 11-12 and figure 3a.

3. The authors mentioned that "Cpd17, but not PF8380, strongly inhibited the ATX+LPC(LPA)-induced RhoA activation in LX2 cells (Fig. 5b)." However, it is not clear what the difference is between groups of ATX+LPC and LPA in figure 5.

We apologise that this was unclear. In the LPA group, only the signalling LPA was added, without any ATX. In the ATX+LPC group, the signalling LPA is enzymatically produced by ATX. While both these groups result in LPA receptor activation, the two groups are not equivalent, as we also discuss in more depth in a recent preprint (https://www.biorxiv.org/content/10.1101/2022.04.09.487723v1). In the context of this figure, the relevant result is that the inhibitor effect in comparison to the ATX+LPC group; the LPA is simply shown as an additional control.

4. The same staining was not quantified in Figure 6b but quantified in Figure 7c, 7e and 7f. The authors should be consistent with the quantification.

We have now quantified all staining's from figure 6 and made the figures 6 and 7 more consistent in terms of presentation.

5. The authors should improve the staining picture quality and background, especially the strong red positive reaction in the H&E staining in Figure 6b is very strange.

We have now improved the quality of images (which was changed while converting the files from PowerPoint to pdf during submission).

6. It is too simplistic to assess only the phenotype in in vivo studies, and authors should also evaluate the RhoA signaling pathway in vivo to confirm conclusion from in vitro studies.

We welcome the suggestion to evaluate Rho signalling also in vivo and have included RhoA (as well as pERK and pAKT) in vivo studies in our response. We do note however, that different cell types respond differently to LPA-mediated Rho signalling (e.g., depending on the expression of different LPA receptors) and that the time-dependent component of Rho signalling is hard to assess in vivo. We used the pMLC2 antibody as a reporter of RhoA activity. We found an increase in MCD-fed mice and a reduction upon Cpd17 treatment; however, these results were not statistically significant. Still, these data (supplementary Fig. 5, page 19, line 8-12 and page 21, line 7-9) corroborate our in vitro observations.

9th Jun 2022

Dear Dr. Bansal,

Thank you for the submission of your revised manuscript to EMBO Molecular Medicine. I am pleased to inform you that we will be able to accept your manuscript pending the following final amendments:

1) Please address the point raised by the referee #2 by adding dose dependent analysis of Cpd17 or by a discussion.
2) Source data: During a standard image analysis we detected potential aberrations in the figure set, and we would like to clarify these issues before accepting your manuscript for publication. We kindly invite you to check western blot images in Figure 5C and Figure S3B, and to send us the related source data. If you make changes to the figure, please include a point-by-point describing what you changed. Furthermore, we encourage you to include the source data for figure panels that show essential data. Numerical data should be provided as individual .xls or .csv files (including a tab describing the data). For blots or microscopy, uncropped images should be submitted (using a zip archive if multiple images need to be supplied for one panel). Please check "Author Guidelines" for more information:
https://www.embopress.org/page/journal/17574684/authorguide#sourcedata
3) Manuscript format: Please reformat your manuscript according to our guidelines outlined here:
https://www.embopress.org/page/journal/17574684/authorguide#researcharticleguide
4) Figures: Please submit all supplementary figures as 5 EV Figures (fuse suppl. figures 5 and 6). Upload individual, high-resolution figure files for EV Figures and add EV figure legends at the end of the main manuscript text. Update the figure callouts in the main text accordingly (callouts for suppl. figure 1 is currently missing). Please check "Author Guidelines" for more information:
https://www.embopress.org/page/journal/17574684/authorguide#figureformat
https://www.embopress.org/page/journal/17574684/authorguide#expandedview
5) In the main manuscript file, please do the following:
- Correct/answer the track changes suggested by our data editors by working from the attached document.
- Limit keywords to max. 5.
- Add author contributions.
- In M&M, add statistical paragraph that should reflect all information that you have filled in the Authors Checklist, especially regarding randomization, blinding, replication.
- Indicate in legends exact n= and exact p= values, not a range, along with the statistical test used. To keep the figures "clear" some authors found providing an Appendix table Sx with all exact p-values preferable. You are welcome to do this if you want to. Please be reminded to show raw values and avoid statistical analyses when n=2. Please check our Author Guidelines:
https://www.embopress.org/page/journal/17574684/authorguide#statisticalanalysis
- Please rename "Conflict of Interest" to "Disclosure Statement & Competing Interests". We updated our journal's competing interests policy in January 2022 and request authors to consider both actual and perceived competing interests. Please review the policy https://www.embopress.org/competing-interests and update your competing interests if necessary.
- Correct the reference citation in the text and reference list. In the text, a reference should be cited by author and year of publication. Include a space between a word and the opening parenthesis of the reference that follows. In the reference list, citations should be listed in alphabetical order. Where there are more than 10 authors on a paper, 10 will be listed, followed by "et al.". Please check "Author Guidelines" for more information.
https://www.embopress.org/page/journal/17574684/authorguide#referencesformat
- Provide data availability statement. If no data are deposited in public repositories, please add the sentence: "This study includes no data deposited in external repositories".

Please check "Author Guidelines" for more information.
https://www.embopress.org/page/journal/17574684/authorguide#availabilityofpublishedmaterial
6) Appendix: Please submit tables currently in supplementary tables file as an "Appendix" with a table of content. Rename "Supplementary Table 1" to "Appendix Table S1" etc. and update the callouts in the main text accordingly.
7) Author contributions: Please specify author contributions in our submission system. CRediT has replaced the traditional author contributions section because it offers a systematic machine readable author contributions format that allows for more effective research assessment. You are encouraged to use the free text boxes beneath each contributing author's name to add specific details on the author's contribution. More information is available in our guide to authors:
https://www.embopress.org/page/journal/17574684/authorguide#authorshipguidelines
8) The Paper Explained: Please provide "The Paper Explained" and add it to the main manuscript text. Please check "Author Guidelines" for more information. https://www.embopress.org/page/journal/17574684/authorguide#researcharticleguide
9) Synopsis: Every published paper now includes a 'Synopsis' to further enhance discoverability. Synopses are displayed on the journal webpage and are freely accessible to all readers. They include separate synopsis image and synopsis text.
- Synopsis image: Please provide a striking image or visual abstract as a high-resolution jpeg file 550 px-wide x (250-400)-px high to illustrate your article.
- Synopsis text: Please provide a short standfirst (maximum of 300 characters, including space) as well as 2-5 one sentence bullet points that summarise the paper as a .doc file. Please write the bullet points to summarise the key NEW findings. They

should be designed to be complementary to the abstract - i.e. not repeat the same text. We encourage inclusion of key acronyms and quantitative information (maximum of 30 words / bullet point). Please use the passive voice.
- Please check your synopsis text and image before submission with your revised manuscript. Please be aware that in the proof stage minor corrections only are allowed (e.g., typos).
10) For more information: Please remove corresponding author's e-mail address. This space should be used to list relevant web links for further consultation by our readers. Could you identify some relevant ones and provide such information as well? Some examples are patient associations, relevant databases, OMIM/proteins/genes links, author's websites, etc...
11) As part of the EMBO Publications transparent editorial process initiative (see our Editorial at http://embomolmed.embopress.org/content/2/9/329), EMBO Molecular Medicine will publish online a Review Process File (RPF) to accompany accepted manuscripts. This file will be published in conjunction with your paper and will include the anonymous referee reports, your point-by-point response and all pertinent correspondence relating to the manuscript. Let us know whether you agree with the publication of the RPF and as here, if you want to remove or not any figures from it prior to publication. Please note that the Authors checklist will be published at the end of the RPF.
12) Please provide a point-by-point letter INCLUDING my comments as well as the reviewer's reports and your detailed responses (as Word file).

I look forward to reading a new revised version of your manuscript as soon as possible.

Yours sincerely,

Zeljko Durdevic

Zeljko Durdevic
Editor
EMBO Molecular Medicine

***** Reviewer's comments *****

Referee #1 (Remarks for Author):

the authors have answers my questions.

Referee #2 (Remarks for Author):

Revised version become better one. I recommend author to add dose dependent analysis of Cpd17. That is important.

Referee #3 (Comments on Novelty/Model System for Author):

The authors used two models of liver disease to make their conclusions more convincing.

Referee #3 (Remarks for Author):

The authors have carefully addressed the reviewer's comments and improved the quality of the manuscript. I have no further comments.

***** Reviewer's comments *****

Referee #1 (Remarks for Author): The authors have answered my questions.

Referee #2 (Remarks for Author): Revised version become better one. I recommend author to add dose dependent analysis of Cpd17. That is important.

Response: In response to this comment, the following has been now included in the discussion section of the manuscript: "Initial studies were performed with different concentrations (100nM to 5µM) of Cpd17 based on which optimal concentration of 1µM was selected for further in vitro studies which also aligned with previous reports where other ATX inhibitors have been tested in vitro (Castelino et al. 2016; Ho et al. 2020)". Please refer to page no. 13, line no. 18-21.

Referee#3 (Comments on Novelty/Model System for Author): The authors used two models of liver disease to make their conclusions more convincing.

Referee #3 (Remarks for Author): The authors have carefully addressed the reviewer's comments and improved the quality of the manuscript. I have no further comments.

We are pleased to inform you that your manuscript is accepted for publication and is now being sent to our publisher to be include signed Open Access Agreement form must be received before the article can be published online.

EMBO Press Author Checklist

Corresponding Author Name: Ruchi Bansal	
Journal Submitted to: EMBO Molecular Medicine	
Manuscript Number: EMM-2022-16333	

USEFUL LINKS FOR COMPLETING THIS FORM
The EMBO Journal - Author Guidelines
EMBO Reports - Author Guidelines
Molecular Systems Biology - Author Guidelines
EMBO Molecular Medicine - Author Guidelines

Reporting Checklist for Life Science Articles (updated January 2022)
This checklist is adapted from Materials Design Analysis Reporting (MDAR) Checklist for Authors. MDAR establishes a minimum set of requirements in transparent reporting in the life sciences (see Statement of Task: 10.31222/osf.io/9sm4x). Please follow the journal's guidelines in preparing your manuscript.
Please note that a copy of this checklist will be published alongside your article.

Abridged guidelines for figures
1. Data
The data shown in figures should satisfy the following conditions:
- the data were obtained and processed according to the field's best practice and are presented to reflect the results of the experiments in an accurate and unbiased manner.
- ideally, figure panels should include only measurements that are directly comparable to each other and obtained with the same assay.
- plots include clearly labeled error bars for independent experiments and sample sizes. Unless justified, error bars should not be shown for technical replicates.
- if n<5, the individual data points from each experiment should be plotted. Any statistical test employed should be justified.
- Source Data should be included to report the data underlying figures according to the guidelines set out in the authorship guidelines on Data Presentation.

2. Captions
Each figure caption should contain the following information, for each panel where they are relevant:
- a specification of the experimental system investigated (eg cell line, species name).
- the assay(s) and method(s) used to carry out the reported observations and measurements.
- an explicit mention of the biological and chemical entity(ies) that are being measured.
- an explicit mention of the biological and chemical entity(ies) that are altered/varied/perturbed in a controlled manner.
- the exact sample size (n) for each experimental group/condition, given as a number, not a range;
- a description of the sample collection allowing the reader to understand whether the samples represent technical or biological replicates (including how many animals, litters, cultures, etc.).
- a statement of how many times the experiment shown was independently replicated in the laboratory.
- definitions of statistical methods and measures:
  - common tests, such as t-test (please specify whether paired vs. unpaired), simple χ2 tests, Wilcoxon and Mann-Whitney tests, can be unambiguously identified by name only, but more complex techniques should be described in the methods section;
  - are tests one-sided or two-sided?
  - are there adjustments for multiple comparisons?
  - exact statistical test results, e.g., P values = x but not P values < x;
  - definition of 'center values' as median or average;
  - definition of error bars as s.d. or s.e.m.

Please complete ALL of the questions below.
Select "Not Applicable" only when the requested information is not relevant for your study.

Materials

Newly Created Materials	Information included in the manuscript?	In which section is the information available? (Reagents and Tools Table, Materials and Methods, Figures, Data Availability Section)
New materials and reagents need to be available; do any restrictions apply?	Not Applicable	

Antibodies	Information included in the manuscript?	In which section is the information available? (Reagents and Tools Table, Materials and Methods, Figures, Data Availability Section)
For **antibodies** provide the following information:<br>- Commercial antibodies: RRID (if possible) or supplier name, catalogue number and or/clone number<br>- Non-commercial: RRID or citation	Yes	Supplementary material: Supplementary table 1 and 2

DNA and RNA sequences	Information included in the manuscript?	In which section is the information available? (Reagents and Tools Table, Materials and Methods, Figures, Data Availability Section)
**Short novel DNA or RNA including primers, probes**: provide the sequences.	Yes	Supplementary material: Supplementary table 3

Cell materials	Information included in the manuscript?	In which section is the information available? (Reagents and Tools Table, Materials and Methods, Figures, Data Availability Section)
**Cell lines:** Provide species information, strain. Provide accession number in repository **OR** supplier name, catalog number, clone number, and/**OR** RRID.	Yes	Materials and Methods section: Cell Lines
**Primary cultures:** Provide species, strain, sex of origin, genetic modification status.	Not Applicable	
Report if the cell lines were recently **authenticated** (e.g., by STR profiling) and tested for mycoplasma contamination.	Yes	Materials and Methods section: Cell Lines

Experimental animals	Information included in the manuscript?	In which section is the information available? (Reagents and Tools Table, Materials and Methods, Figures, Data Availability Section)
**Laboratory animals or Model organisms:** Provide species, strain, sex, age, genetic modification status. Provide accession number in repository **OR** supplier name, catalog number, clone number, **OR** RRID.	Yes	Materials and Methods section: Animal Experiments
**Animal observed in or captured from the field:** Provide species, sex, and age where possible.	Not Applicable	
Please detail **housing and husbandry conditions**.	Yes	Materials and Methods section: Animal Experiments

Plants and microbes	Information included in the manuscript?	In which section is the information available? (Reagents and Tools Table, Materials and Methods, Figures, Data Availability Section)
**Plants:** provide species and strain, ecotype and cultivar where relevant, unique accession number if available, and source (including location for collected wild specimens).	Not Applicable	
**Microbes:** provide species and strain, unique accession number if available, and source.	Not Applicable	

Human research participants	Information included in the manuscript?	In which section is the information available? (Reagents and Tools Table, Materials and Methods, Figures, Data Availability Section)
If collected and within the bounds of privacy constraints report on age, sex and gender or ethnicity for all study participants.	Not Applicable	

Core facilities	Information included in the manuscript?	In which section is the information available? (Reagents and Tools Table, Materials and Methods, Figures, Data Availability Section)
If your work benefited from core facilities, was their service mentioned in the acknowledgments section?	Not Applicable	

Design

Study protocol	Information included in the manuscript?	In which section is the information available? (Reagents and Tools Table, Materials and Methods, Figures, Data Availability Section)
If study protocol has been **pre-registered, provide DOI in the manuscript**. For clinical trials, provide the trial registration number **OR** cite DOI.	Not Applicable	
Report the **clinical trial registration number** (at ClinicalTrials.gov or equivalent), where applicable.	Not Applicable	

Laboratory protocol	Information included in the manuscript?	In which section is the information available? (Reagents and Tools Table, Materials and Methods, Figures, Data Availability Section)
Provide DOI OR other citation details if **external detailed step-by-step protocols** are available.	Not Applicable	

Experimental study design and statistics	Information included in the manuscript?	In which section is the information available? (Reagents and Tools Table, Materials and Methods, Figures, Data Availability Section)
Include a statement about **sample size** estimate even if no statistical methods were used.	Yes	Materials and Methods
Were any steps taken to minimize the effects of subjective bias when allocating animals/samples to treatment (e.g. **randomization procedure**)? If yes, have they been described?	Yes	Materials and Methods
Include a statement about **blinding** even if no blinding was done.	Yes	Materials and Methods
Describe **inclusion/exclusion criteria** if samples or animals were excluded from the analysis. Were the criteria pre-established?<br><br>If sample or data points were omitted from analysis, report if this was due to attrition or intentional exclusion and provide justification.	Yes	Materials and Methods
For every figure, are **statistical tests** justified as appropriate? Do the data meet the assumptions of the tests (e.g., normal distribution)? Describe any methods used to assess it. Is there an estimate of variation within each group of data? Is the variance similar between the groups that are being statistically compared?	Yes	Materials and Methods, and respective figure legends

Sample definition and in-laboratory replication	Information included in the manuscript?	In which section is the information available? (Reagents and Tools Table, Materials and Methods, Figures, Data Availability Section)
In the figure legends: state number of times the experiment was **replicated** in laboratory.	Yes	Materials and Methods
In the figure legends: define whether data describe **technical or biological replicates**.	Yes	Materials and Methods

Ethics

Ethics	Information included in the manuscript?	In which section is the information available? (Reagents and Tools Table, Materials and Methods, Figures, Data Availability Section)
Studies involving **human participants**: State details of **authority granting ethics approval** (IRB or equivalent committee(s), provide reference number for approval.	Not Applicable	
Studies involving **human participants**: Include a statement confirming that **informed consent** was obtained from all subjects and that the experiments conformed to the principles set out in the WMA Declaration of Helsinki and the Department of Health and Human Services Belmont Report.	Yes	Materials and Methods
Studies involving **human participants:** For publication of **patient photos**, include a statement confirming that consent to publish was obtained.	Not Applicable	
Studies involving experimental **animals**: State details of **authority granting ethics approval** (IRB or equivalent committee(s), provide reference number for approval. Include a statement of compliance with ethical regulations.	Yes	Materials and Methods
Studies involving **specimen and field samples:** State if relevant **permits** obtained, provide details of authority approving study; if none were required, explain why.	Not Applicable	

Dual Use Research of Concern (DURC)	Information included in the manuscript?	In which section is the information available? (Reagents and Tools Table, Materials and Methods, Figures, Data Availability Section)
Could your study fall under dual use research restrictions? Please check biosecurity documents and list of **select agents and toxins** (CDC): https://www.selectagents.gov/sat/list.htm	Not Applicable	
If you used a select agent, is the security level of the lab appropriate and reported in the manuscript?	Not Applicable	
If a study is subject to dual use research of concern regulations, is the name of the **authority granting approval and reference number** for the regulatory approval provided in the manuscript?	Not Applicable	

Reporting

The MDAR framework recommends adoption of discipline-specific guidelines, established and endorsed through community initiatives. Journals have their own policy about requiring specific guidelines and recommendations to complement MDAR.

Adherence to community standards	Information included in the manuscript?	In which section is the information available? (Reagents and Tools Table, Materials and Methods, Figures, Data Availability Section)
State if relevant guidelines or checklists (e.g., **ICMJE, MIBBI, ARRIVE, PRISMA**) have been followed or provided.	Not Applicable	
For **tumor marker prognostic studies,** we recommend that you follow the **REMARK** reporting guidelines (see link list at top right). See author guidelines, under 'Reporting Guidelines'. Please confirm you have followed these guidelines.	Not Applicable	
For **phase II and III randomized controlled trials**, please refer to the **CONSORT** flow diagram (see link list at top right) and submit the CONSORT checklist (see link list at top right) with your submission. See author guidelines, under 'Reporting Guidelines'. Please confirm you have submitted this list.	Not Applicable	

Data Availability

Data availability	Information included in the manuscript?	In which section is the information available? (Reagents and Tools Table, Materials and Methods, Figures, Data Availability Section)
Have **primary datasets** been deposited according to the journal's guidelines (see 'Data Deposition' section) and the respective accession numbers provided in the Data Availability Section?	Not Applicable	
Were **human clinical and genomic datasets** deposited in a public access-controlled repository in accordance to ethical obligations to the patients and to the applicable consent agreement?	Not Applicable	
Are **computational models** that are central and integral to a study available without restrictions in a machine-readable form? Were the relevant accession numbers or links provided?	Not Applicable	
If publicly available data were reused, provide the respective **data citations in the reference list**.	Not Applicable	
